# Validation of an Algorithmic Pipeline for Wrist-Worn Devices to Estimate Walking Speed in People with Multiple Long-Term Conditions

**DOI:** 10.3390/s26154946

**Published:** 2026-08-05

**Authors:** Dimitrios Megaritis, Lisa Alcock, Kirsty Scott, Hugo Hiden, Ioannis Vogiatzis, Silvia Del Din

**Affiliations:** 1 Faculty of Health and Life Sciences, Northumbria University Newcastle, Newcastle upon Tyne NE1 8ST, UK; ioannis.vogiatzis@northumbria.ac.uk; 2 Translational and Clinical Research Institute, Newcastle University, Newcastle upon Tyne NE4 5PL, UK; lisa.alcock@newcastle.ac.uk (L.A.); kirsty.scott-singer@newcastle.ac.uk (K.S.); silvia.del-din@newcastle.ac.uk (S.D.D.); 3 National Institute for Health and Care Research (NIHR), Newcastle Biomedical Research Centre (BRC), Newcastle University, Newcastle upon Tyne NE4 5PL, UK; 4 School of Computing, Newcastle University, Newcastle upon Tyne NE4 5TG, UK; hugo.hiden@newcastle.ac.uk

**Keywords:** wrist-worn devices, digital biomarkers, digital mobility outcomes, gait analysis, multiple long-term conditions

## Abstract

**Highlights:**

**What are the main findings?**

- A modular pipeline for wrist-worn devices achieved walking speed accuracy (MAE: 0.13 m/s, near-zero bias) comparable to lower-back reference systems in older adults aged 65–90 years with complex multimorbidity.
- Gait sequence detection performed robustly (recall: 0.90), while initial contact detection (recall: 0.77) was identified as the main bottleneck, limiting downstream cadence estimation due to the inherent variability of non-standardised wrist swing.

**What are the implications of the main findings?**

- This technically validated wrist-worn pipeline for digital mobility outcomes in people with multiple long-term conditions establishes a benchmark for accessible, real-world gait assessment in this high-burden population.
- The validated, open-source pipeline provides a foundation for real-world deployment and future development of novel gait analysis approaches.

**Abstract:**

Wrist-worn devices offer a practical means of monitoring gait, yet no validated end-to-end pipeline exists for deriving digital mobility outcomes (DMOs), including cadence, stride length (SL), and walking speed (WS), in people with multiple long-term conditions (MLTC, the coexistence of two or more long-term conditions). This study presents the first modular pipeline for wrist-worn devices for DMO estimation, validated in 45 older adults with MLTC (65–90 years), whose conditions spanned four multimorbidity clusters (cardiometabolic, painful conditions, pulmonary, and cancer), across laboratory tasks, using stereophotogrammetry as the reference. Algorithms were selected independently for each block (gait sequence detection (GSD), initial contact detection (ICD), SL) using novel, fine-tuned, and adaptive versions of established methods developed on an independent cohort. Blocks were first validated independently before being integrated into a pipeline capturing cumulative error propagation. GSD achieved a recall of 0.90. ICD was robust across algorithms, with the best-performing algorithm achieving a recall of 0.76 and precision of 0.82. At the pipeline level, the best-performing pipeline achieved an SL error of 0.14 m with near-zero bias, cadence absolute error of 7.71 steps/min, and WS absolute error of 0.13 m/s. These findings support wrist-worn devices for objective gait assessment in multimorbid populations, establishing a validated open-source pipeline for real-world deployment.

## 1. Introduction

Digital mobility outcomes (DMOs) have emerged as promising tools for the objective assessment of real-world mobility, with accumulating evidence linking real-world mobility performance to broader health status, and disease severity [1,2,3,4]. A critical prerequisite for their adoption as health biomarkers in clinical trials and routine care is robust technical validation [5], demonstrating accuracy and reliability across the full estimation pipeline rather than individual algorithmic components in isolation [6].

However, existing validation studies share critical limitations. First, they have focused on single long-term conditions, despite older adults commonly presenting with multiple long-term conditions that often span several disease domains and are associated with heterogeneous gait impairments [7,8]. Second, pipeline validations have assessed individual components (i.e., blocks) using reference-system inputs, rather than the full wearable pipeline, where errors accumulate across stages. In our previous work, we validated gait sequence detection (GSD), initial contact detection (ICD), and stride length (SL) in individuals with a mild-to-moderate burden of multiple long-term conditions (MLTC), defined as the coexistence of two or more long-term conditions [9]. During that work, we developed and validated novel wrist-adapted, fine-tuned versions of state-of-the-art algorithms specifically designed to accommodate the signal complexity of wrist-worn devices in populations with MLTC, providing the methodological foundation for the present study. Third, no study has yet derived composite digital mobility outcomes, including cadence (CAD), stride length, and walking speed (WS), within a fully integrated end-to-end pipeline operating on raw wrist-worn data in free-living conditions.

Despite widespread adoption of wrist technology, the above limitations represent a fundamental gap between accessibility and the technical validity required before these solutions can support clinical application.

The present study addresses all three limitations simultaneously, by presenting, to the authors’ knowledge, the first wrist-derived composite DMO pipeline and comprehensively validating it in individuals with a high clinical burden of MLTC. Specifically, we aimed to achieve the following:

(i) Validate and rank GSD algorithms in a highly representative sample of individuals with MLTC, extending our previous findings as a baseline for downstream pipeline validation;
(ii) Validate all pipeline combinations for ICD, SL, cadence, and walking speed across all algorithmic combinations, establishing a best-performing pipeline;
(iii) Assess pipeline validity at the individual participant level, identifying which pipeline configurations perform best for each participant and whether performance varies systematically across the multimorbid sample.

## 2. Materials and Methods

### 2.1. Study Population

Fifty participants with MLTC were recruited from primary- and secondary-care clinics, as well as community-based health charities, as part of the MultiMobility study (ISRCTN25008143, NCT06473168). Eligible participants were adults aged ≥ 65 years who were able to provide written informed consent, could read and write in English, and were mobile (including those using walking aids). Inclusion required at least two co-occurring long-term conditions of equivalent clinical significance from a predefined list spanning the cardiovascular, metabolic, respiratory, musculoskeletal, and neurological domains, including: arthritis, asthma, atrial fibrillation, bronchiectasis, cancer, chronic kidney disease, chronic obstructive pulmonary disease, coronary heart disease, anxiety, depression, diabetes mellitus, heart failure, hypertension, osteoporosis, Parkinson’s disease, peripheral vascular disease, and stroke or transient ischaemic attack. Individuals were excluded if they were unable to provide informed consent, had severe mental health problems, or had active malignancy requiring chemotherapy, radiotherapy, or urgent surgery. Written informed consent was obtained from all participants prior to participation. Approval was granted by the NHS Health Research Authority (NHS Health Research Authority North East York Research Ethics Committee; IRAS: 340676), and the Newcastle upon Tyne NHS Healthcare Foundation Trust (R&D 10877).

### 2.2. Experimental Protocol

Participants completed a laboratory-based assessment comprising eight motor tasks of increasing complexity: straight walking at preferred (comfortable), fast, and slow speeds; Timed Up and Go (TUG); L Test; Surface Test; Continuous Walking Test (60 s); and Simulated Daily Activities (SDA) [10]. Task descriptions followed the Mobilise-D protocol [10] with modifications to accommodate frailer and slower-walking individuals with MLTC [10], and are detailed as follows:

- Straight Walking (Slow, Comfortable, Fast): Participants walked 5 m from a standing start at three self-selected speeds (comfortable, slow, fast, in this standardised order), each repeated twice for reproducibility.
- Timed Up and Go (TUG): Participants rose from a chair, walked 3 m at a comfortable pace, turned 180°, returned, and sat down.
- L Test: Participants rose from a chair, walked straight at a comfortable pace, turned 90° around a cone, walked to a second cone, turned 180°, then made a final 90° turn to return to the chair.
- Hallway Test: Participants walked around a defined circuit twice (approximately 20 m).
- Continuous Walking Test (CWT): Participants walked continuously at a comfortable pace for 60 s around a 10 × 10 m square circuit.
- Simulated Daily Activities (SDA): Participants performed a series of daily living tasks while moving around the room from a seated starting position, simulating real-world transitions.

### 2.3. Inertial Measurement Unit Data

Wrist IMU data were collected using an Axivity AX6 sensor (23 × 32.5 × 8.9 mm, 11 g, Axivity Ltd., Newcastle, UK) placed on the non-dominant wrist using an elastic strap. The device recorded triaxial acceleration and gyroscope data at a sampling frequency of 100 Hz, with a 16-bit resolution. The accelerometer range was configured to ±8× *g* and the gyroscope range to ±2000 degrees per second (dps), providing full 6-degrees-of-freedom motion data.

### 2.4. Reference Data

Reference spatiotemporal gait parameters were obtained using a stereophotogrammetry (SP) system (100 Hz), serving as the gold standard for laboratory-based tasks. The SP system, comprising 14 Vicon Bonita cameras (100 Hz), reconstructs the 3D trajectories of reflective markers attached to the feet and lower back, with systematic and random errors minimised to a few millimetres via calibration and filtering [10,11]. A quality-control spot-check procedure was performed for each marker acquisition following the methodology described in [10,11]; it reported systematic tracking errors below 2.5 mm. A bespoke marker set was adopted following the procedures described previously, including four markers on each foot for gait event detection [10]. Two of the four markers on each foot were positioned asymmetrically relative to the others to facilitate automatic marker-label recognition during data processing [10]. Walking bouts were defined as continuous sequences containing at least two consecutive strides of both feet, with consecutive bouts separated by breaks greater than 3 s [12]. IMU and reference system data were synchronised using timestamps referred to a common clock. Synchronisation was additionally verified by comparing the movement of reflective markers placed on the wrist device with the corresponding IMU signal. A maximum synchronisation error of ±10 ms was considered acceptable, as this level of accuracy has previously been reported for similar setups [10,11].

### 2.5. Algorithms

The algorithms used for GSD, ICD, and SL estimation are described in detail in our previous work [9] and are openly available in our implementation (multigait, v1.0) [13]. All algorithms are novel wrist-adapted, fine-tuned, and adaptive variants of established gait algorithms, specifically developed and optimised for wrist-worn IMUs in multimorbid populations using an independent development cohort, with no overlap between the development cohort and the present validation cohort.

For the present study, GSD algorithms were evaluated and ranked in Aim 1. ICD, SL, cadence, and walking speed were evaluated as pipeline components in Aim 2, using wearable-derived inputs throughout. ICD performance was evaluated exclusively on true-positive walking bouts, as wearable-derived gait sequence indices require alignment with reference walking bout indices to enable meaningful comparison of initial contact events. In terms of the composite DMOs, cadence was derived from initial contacts using a per-second cadence estimation algorithm based on inter-contact intervals with Hampel filtering for outlier suppression. Walking speed was computed per walking bout as the product of cadence and stride length, following Kirk et al. [6]:

Walking speed [m/s]=(cadence [steps/min]/(2×60))×stride length [m]

All algorithms and the full pipeline, which extracts 40 DMOs as weekly aggregates, daily aggregates, and WB level DMOs across walking bouts of varying duration (all WBs, 10–30 s, >10 s, >30 s, >60 s), are publicly available as a Python package (PyPi) and on GitHub (multigait, v1.0) [13]. Beyond standard mean-level gait outcomes (walking speed, stride length, cadence) and accumulated mobility outcomes (number and duration of WBs, steps during WBs, total walking time), this includes within-bout variability measures for walking speed, cadence, and stride length using the coefficient of variation and, to our knowledge novel to gait analysis, the root mean square of successive differences (RMSSD), previously used mainly in heart rate variability, which we hypothesise may capture novel information on gait variability.

### 2.6. Pipeline Construction

**Figure 1:**
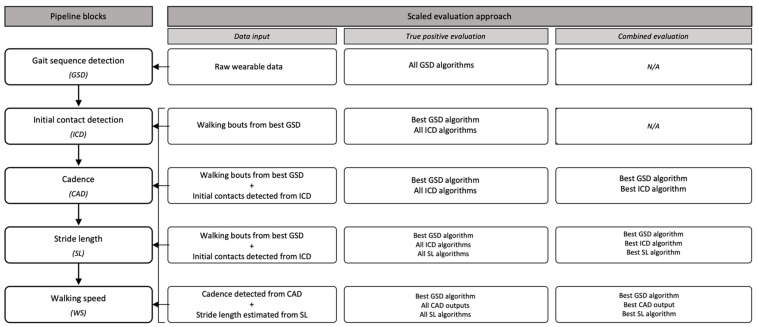
Visual presentation of the pipeline and validation structure. Pipeline blocks: sequential algorithmic blocks. Data input: wearable-derived inputs at each stage. True-positive evaluation: algorithms assessed on walking bouts detected by both systems (≥85% overlap). Combined evaluation: algorithms assessed on mean DMO values across all detected bouts.

The full wearable pipeline cascade is sequentially similar to the MobGap pipeline [14]: GSD detects walking bouts; ICD detects initial contacts within those bouts; SL and cadence are estimated from the detected contacts; walking speed is derived from cadence and stride length. Pseudocode summarising this pipeline process is provided in the Supplementary Material (Algorithm S1). A key methodological novelty of this study is the adoption of a scaled pipeline evaluation approach, in which algorithms are introduced and assessed progressively at each stage using only wearable-derived inputs. GSD was first evaluated independently against the reference system to identify the best-performing algorithm. ICD was then evaluated within the wearable pipeline, using detected walking bouts from the best GSD as input, thereby assessing pipeline rather than standalone algorithmic validity. SL and cadence were subsequently evaluated using the wearable GSD and all ICD combinations, and walking speed was derived from the full cascade of wearable-derived GSD, ICD, cadence, and stride length. This progressive approach ensures that errors accumulate naturally through the pipeline at each stage, providing a realistic assessment of end-to-end pipeline performance (Figure 1).

### 2.7. Sample Size

The required number of walking bouts for robust estimation of the intraclass correlation coefficient (ICC) was calculated using the Bonett et al. approximation [15]. Based on an expected ICC of 0.64 from the best-performing GSD algorithm from our previous work [5], and a desired 95% confidence interval width of 0.2, approximately 100 walking bouts are needed to achieve sufficient precision.

### 2.8. Validation Approach

All pipeline inputs are wearable-derived, with no reference system inputs at any stage. A total of 27 pipeline combinations were evaluated for SL (9 ICD × 3 SL), 9 for cadence (9 ICD × 1 cadence algorithm), and 27 for walking speed (9 ICD × 3 SL). Two complementary evaluation approaches were employed, following Kirk et al. [6]. The true-positive evaluation served as the primary analysis for full ranking and selection of all algorithms and pipeline combinations. The combined evaluation was applied exclusively to the best-performing pipeline, identified through the true-positive evaluation, for verification and error quantification (Figure 1).

True-positive evaluation: Only walking bouts detected by both systems with an overlap exceeding 85% were compared, enabling paired comparisons including ICC and Bland–Altman analysis. The 85% threshold yielded 145 true-positive walking bouts, exceeding the pre-specified minimum of 100 required for robust statistical power. The sensitivity of the performance index to the TP threshold was additionally examined across a range of values (0.50–0.95) for cadence, stride length, and walking speed.

Combined evaluation: Mean DMO values were calculated across all detected walking bouts per participant per task, providing a traditional evaluation approach independent of the true-positive evaluation.

### 2.9. Performance Metrics and Ranking

Performance metrics and the ranking methodology follow previous work [9,12], with the novel addition of pipeline-level ranking for cadence and walking speed. Pseudocode summarising the performance index calculation and ranking procedure is provided in the Supplementary Material (Algorithm S2). For GSD, the performance index, metrics, and weights are described in detail in Megaritis et al. [9] and summarised in Table S1. For ICD and SL evaluated within the pipeline context, the same weighting frameworks are applied [9] (Tables S2 and S3, respectively).

For cadence, the same weighting framework was applied [9], with the exception that absolute cadence error was normalised using min–max rather than exponential normalisation, as the unit scale of cadence (steps/min) renders the latter insensitive to meaningful differences between algorithms (Table S4). For walking speed, a novel performance index was developed comprising ICC(2,1), MAE, MARE, and limits of agreement (LoA), with the weights presented in Table S5. All other error metrics were normalised using exponential normalisation. For presentation, pipeline performance metrics were aggregated across all laboratory tasks by averaging metric values and summing walking bout counts across tests. The full per-test results for all pipeline combinations are presented in the Supplementary Materials. All analyses were performed in Python (version 3.12). The complete data acquisition, synchronisation, and processing workflow described above is summarised in Figure S1 (Supplementary Materials).

### 2.10. Participant-Level Pipeline Analysis

To assess individual-level variation in algorithmic and pipeline validity, a simplified performance index was computed for each participant × algorithm or pipeline combination, averaged across all laboratory tasks. Since population-level metrics, including ICC and limits of agreement, require multiple participants and cannot be meaningfully computed at the individual level, these were excluded; all remaining metrics from the respective DMO performance indices were retained, with weights redistributed proportionally (Tables S1–S5, Supplementary Materials). The algorithms and pipeline combinations were ranked for each participant based on this score. This data-mining approach was applied across GSD (Aim 1) and ICD, SL, cadence, and walking speed (Aim 2) to identify whether systematic variation in validity exists across participants, informing the potential development of personalised analytical pipelines.

For each participant, percentage performance loss was calculated as the difference between the performance index achieved by that participant’s best-performing algorithm or pipeline combination and the performance index achieved, for that same participant, by the algorithm or pipeline identified as best-performing at the population level, expressed as a percentage of the participant’s best-achievable performance index:

Performance loss [%] = [(PIpersonal-best − PIpopulation-best)/PIpersonal-best] × 100

To explore whether their morbidity profile contributed to such variation, participants were assigned to one or more multimorbidity clusters, including cardiometabolic, painful conditions (arthritis, chronic pain, and neurological conditions), pulmonary, and cancer, following the disease categories identified via latent class analysis in the UK Biobank cohort by Steell et al. [16,17] for the oldest age strata (65+/74+), comparable to our sample; performance loss was descriptively compared across participants grouped by number of multimorbidity clusters.

## 3. Results

### 3.1. Participants

The laboratory data were valid for 45 of the 50 recruited participants; inclusion required at least one task with both wearable and reference system data available. The demographics and clinical characteristics are presented in Table 1. The most prevalent domains were cardiometabolic and painful conditions (both *n* = 44, 97%), followed by pulmonary conditions (n = 20, 44%) and cancer (n = 13, 28%). Most participants had conditions spanning multiple MLTC clusters (two clusters: n = 21; three clusters: n = 14; four clusters: n = 8), while only five participants (11%) were assigned to a single cluster.

### 3.2. Algorithmic Validation

#### Gait Sequence Detection

The performance metrics for the GSD algorithms are presented in Table 2. The novel wrist-adapted and fine-tuned Ionescu method [18] achieved the highest performance index (0.72), demonstrating the strongest balance of accuracy (0.85) and recall (0.90) across all the evaluated algorithms. The Iluz method ranked second (PI = 0.69), exhibiting the highest ICC (0.77) and precision (0.86), though with lower specificity (0.72) compared to the top-performing method. Ionescu was therefore selected as the fixed GSD component for all downstream pipeline analyses.

### 3.3. Pipeline True-Positive Validation

#### Initial Contact Detection

The performance metrics for the ICD algorithms evaluated within the wearable pipeline are presented in Table 3. Zijlstra [24] and McCamley [25] achieved the highest performance indices (0.82), with Zijlstra demonstrating the highest precision (0.82) and McCamley the strongest recall (0.77) alongside the lowest absolute timing error (0.12 s). Given the narrow performance range across algorithms (PI = 0.79–0.82) and overlapping confidence intervals, all nine ICD algorithms were carried forward for downstream pipeline validation.

### 3.4. Cadence

**Figure 2:**
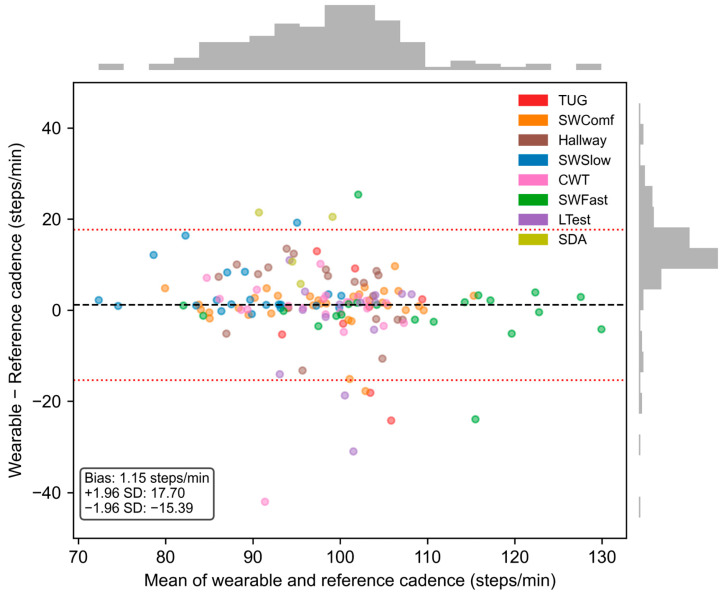
Bland–Altman plot of cadence estimation for the best-performing pipeline (Ionescu GSD + Zijlstra ICD), showing the difference between wearable-derived and reference cadence against their mean across all true-positive walking bouts. The dashed black line represents the mean bias and the dotted red lines represent the 95% limits of agreement. Each point represents one walking bout, colour-coded by laboratory task. SW: straight walking; Comf: comfortable speed; Slow: slow speed; Fast: fast speed; TUG: Timed Up and Go; L Test: L-shaped walking test; CWT: Continuous Walking Test; SDA: Simulated Daily Activities.

The pipeline performance metrics for cadence estimation are presented in Table 4. The Zijlstra-based cadence estimation achieved the highest performance index (0.78), with the lowest absolute error (5.71 steps/min) and highest ICC (0.70), and a minimal overestimation bias of 0.98 steps/min. Notably, while the McCamley ICD method ranked among the top ICD algorithms in isolation, its cadence pipeline performance was lower (0.71), highlighting that ICD accuracy in isolation does not directly translate to cadence estimation accuracy within the full wearable pipeline. The Zijlstra method was therefore identified as the best-performing ICD component for cadence estimation. Figure 2 presents the Bland–Altman plot for cadence estimation of the best-performing pipeline (Ionescu + Zijlstra), illustrating agreement between wearable and reference cadence across all true-positive walking bouts. The pipeline demonstrated a small positive bias of 1.15 steps/min, with limits of agreement of −15.39 to 17.70 steps/min, indicating a slight systematic overestimation of cadence. Errors were broadly distributed around the bias line, with no clear proportional bias across the cadence range.

### 3.5. Stride Length

**Figure 3:**
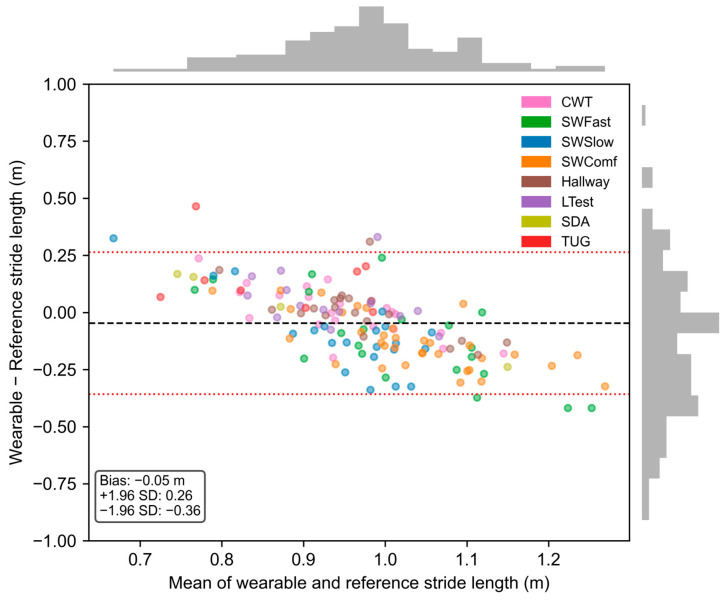
Bland–Altman plot of stride length estimation for the best-performing pipeline (IonescuGSD + PhamIC + WeinbergSL adaptive), showing the difference between wearable-derived and reference stride length against their mean across all true-positive walking bouts. The dashed black line represents the mean bias and the dotted red lines represent the 95% limits of agreement. Each point represents one walking bout, colour-coded by laboratory task. SW: straight walking; Comf: comfortable speed; Slow: slow speed; Fast: fast speed; TUG: Timed Up and Go; L Test: L-shaped walking test; CWT: Continuous Walking Test; SDA: Simulated Daily Activities.

The SL algorithms were first evaluated in isolation using reference-derived walking bouts and initial contacts (Supplementary Table S6); the three best-performing algorithms, the Weinberg adaptive, Weinberg, and Bylemans adaptive foot-length-augmented versions, were carried forward for pipeline evaluation. These top three algorithms achieved absolute errors ranging from 0.14 to 0.15 m. The pipeline performance metrics for SL estimation across all ICD × SL combinations are presented in Table 5. The Weinberg adaptive version consistently outperformed the standard version, with the Pham combination achieving the highest performance index (0.78), a mean absolute error of 0.14 m, and an ICC of 0.64. Notably, while Zijlstra was the top-performing ICD algorithm for cadence estimation, it ranked second for SL paired with the Weinberg adaptive version (performance index = 0.78, MAE = 0.14 m, ICC = 0.65), representing a negligible difference compared to the top combination and with the second-best-performing method having a similar error. The performance difference across the top combinations was marginal, with performance index values ranging from 0.71 to 0.78 and overlapping confidence intervals across all Weinberg-based pipelines. Figure 3 presents the Bland–Altman plot for stride length estimation of the best-performing pipeline (Ionescu + Zijlstra + Weinberg adaptive version), illustrating agreement between the wearable and reference stride length across all true-positive walking bouts. The pipeline demonstrated a negligible bias of −0.05 m, with limits of agreement of −0.36 to 0.26 m, indicating minimal systematic error. A biphasic pattern is evident, with overestimation at shorter stride lengths and underestimation at longer stride lengths, consistent with the intensity-based nature of the Weinberg algorithm. The stride length relative error decreased with increasing walking speed and walking bout duration, consistent with an exponential decay pattern, with errors particularly elevated at very low walking speeds and stabilising from approximately 0.6 m/s onward, as shown in Figures S1 and S2.

### 3.6. Walking Speed

**Figure 4:**
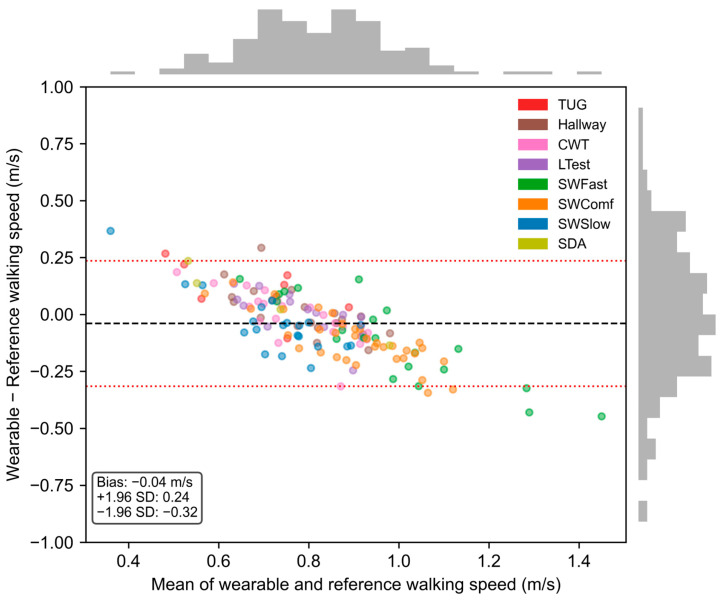
Bland–Altman plot of walking speed estimation for the best-performing pipeline (Ionescu + Zijlstra + Weinberg adaptive), showing the difference between wearable-derived and reference walking speed against the reference walking speed across all true-positive walking bouts. The dashed black line represents the mean bias and the dotted red lines represent the 95% limits of agreement. Each point represents one walking bout, colour-coded by laboratory task. SW: straight walking; Comf: comfortable speed; Slow: slow speed; Fast: fast speed; TUG: Timed Up and Go; L Test: L-shaped walking test; CWT: Continuous Walking Test; SDA: Simulated Daily Activities.

**Figure 5:**
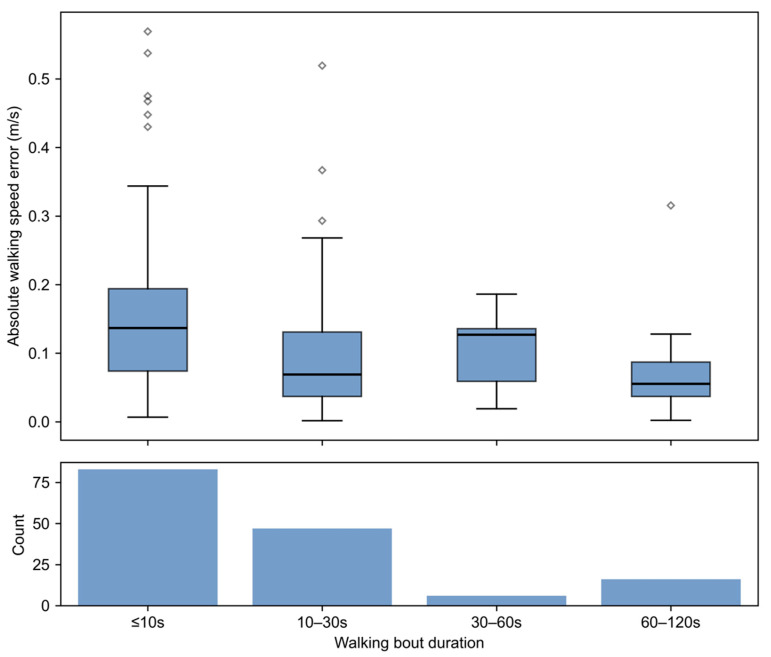
Absolute walking speed error stratified by walking bout duration for the best-performing pipeline (Ionescu + Zijlstra + Weinberg adaptive). Upper panel shows boxplots of absolute error per duration category across all true-positive walking bouts; the horizontal black line represents the median, boxes represent the interquartile range, whiskers extend to 1.5× IQR, and diamonds represent outliers. Lower panel shows the number of true-positive walking bouts in each duration category. Walking bouts exceeding 120 s were excluded.

The pipeline performance metrics for walking speed estimation across all ICD × SL combinations are presented in Table 6. Consistent with the individual DMO rankings, the Zijlstra IC combined with Weinberg SL adaptive achieved the highest performance index (PI = 0.78), with a mean absolute error of 0.12 m/s and an ICC of 0.68, confirming that the best-performing ICD algorithm for cadence and the best-performing SL algorithm together yielded the optimal walking speed pipeline. Performance declined progressively with lower-ranked ICD and SL combinations, with Mico-Amigo IC-based pipelines performing substantially worse regardless of SL algorithm, reaching absolute errors of up to 0.54 m/s. Figure 4 presents the Bland–Altman plot for walking speed estimation of the best-performing pipeline (Ionescu + Zijlstra + Weinberg adaptive), illustrating agreement between wearable and reference walking speed across all true-positive walking bouts. The pipeline demonstrated a negligible bias of −0.04 m/s, with limits of agreement of −0.32 to 0.24 m/s, indicating minimal systematic error. A clear negative proportional bias is evident, with overestimation at slower walking speeds and underestimation at faster walking speeds, consistent with the known speed-dependent behaviour of intensity-based stride length algorithms. Figure 5 presents walking speed absolute error stratified by walking bout duration for the best-performing pipeline. Errors were highest for the shortest walking bouts (≤10 s, median ≈ 0.14 m/s) and decreased progressively with longer bout durations, with the lowest errors observed for bouts between 60 and 120 s (median ≈ 0.06 m/s). The influence of task complexity on walking speed error is presented in Supplementary Figure S4. The sensitivity of the performance index to the TP overlap threshold (0.50–0.95) for cadence, stride length, and walking speed is presented in Supplementary Figure S5. The duration distribution of walking bouts excluded from the true-positive evaluation is presented in Supplementary Figure S6.

### 3.7. Combined Evaluation

The combined evaluation performance metrics for the best-performing modular pipeline are presented in Table 7. The pipeline comprised Ionescu GSD, Zijlstra IC for step detection, cadence, and walking speed, and Weinberg adaptive variant SL throughout. Given that stride length estimation relies on initial contact detection, Pham IC was selected for this component as it demonstrated superior accuracy for SL estimation in the true-positive evaluation, reflecting the modular nature of the pipeline. Scatter plots and regression slopes of wearable-derived versus reference-derived values for cadence, stride length, and walking speed are presented in Supplementary Figure S7.

### 3.8. Participant-Level Pipeline Analysis

**Figure 6:**
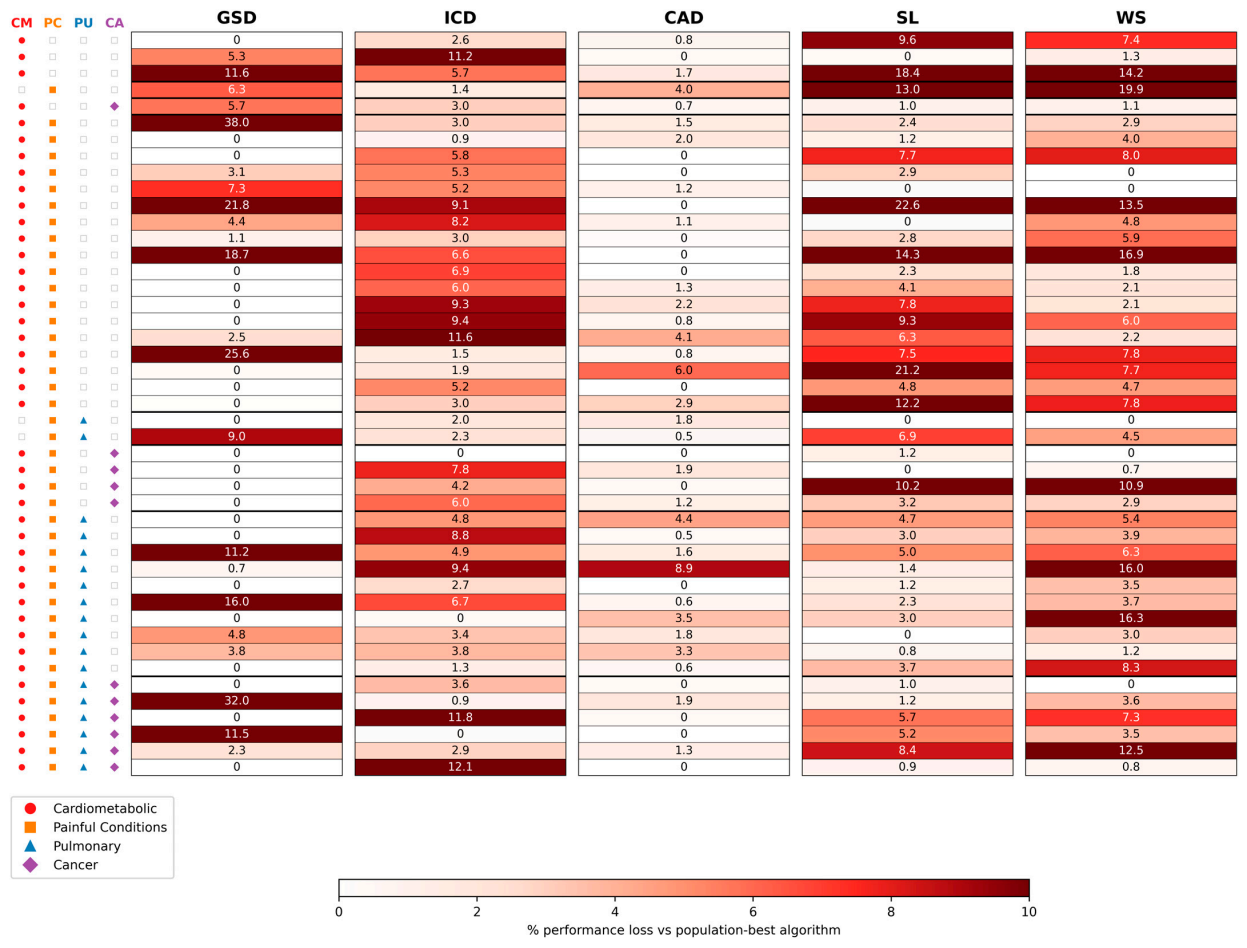
Per-participant performance loss (%) from the population-best modular pipeline (Ionescu GSD; Zijlstra IC for ICD and cadence; Pham IC + Weinberg adaptive SL for stride length; Zijlstra IC + Weinberg adaptive SL for walking speed), expressed as the percentage difference between each participant’s personal-best and population-best algorithm score. Participants are ordered by multimorbidity cluster combination (top to bottom: increasing number of clusters). Colour intensity reflects magnitude of loss (white = 0%, i.e., personal best equals population best, dark red = ≥10%). GSD: gait sequence detection; ICD: initial contact detection; SL: stride length; CAD: cadence; WS: walking speed; CM: cardiometabolic; PC: painful conditions; PU: pulmonary; CA: cancer.

The per-participant performance loss from the population-best modular pipeline across all DMOs is presented in Figure 6, with participants grouped by multimorbidity cluster combination. The population-best pipeline demonstrated low loss across many participants, with a substantial proportion of cells equal to 0%, indicating that the universal pipeline is near-optimal for a large part of the sample. Descriptively, participants with conditions confined to a single multimorbidity cluster showed higher mean performance loss across all DMOs compared with those spanning two, three, or four clusters (Supplementary Table S7), suggesting that the universal pipeline performs best in participants with more complex multimorbidity profiles. Cadence showed consistently low performance loss across all participants and cluster combinations, confirming its robustness as a universal pipeline output. Performance loss for ICD, SL, and WS was more variable across cluster groups, consistent with the population-level finding that initial contact detection and spatial estimation represent the primary sources of pipeline error.

## 4. Discussion

### 4.1. Main Findings

This study presents the first wrist-derived composite DMO pipeline capable of estimating cadence, stride length, and walking speed at the level of individual walking bouts, validated in older adults with multiple long-term conditions. The pipeline adopts a modular design in which the optimal signal processing algorithm is selected independently for each DMO, with Ionescu for GSD; Zijlstra for ICs, cadence, and walking speed; and Pham IC with Weinberg adaptive SL for stride length estimation. All algorithms employed are novel wrist-adapted, fine-tuned versions of established methods developed and optimised in our previous work [9] using an independent sample, with fine-tuning conducted entirely independently of the present validation cohort. This modular approach extends our previous work [9], which validated individual components using reference-fed inputs, to a fully wearable cascade where errors accumulate naturally across pipeline stages. The best-performing pipeline achieved a walking speed MAE of 0.13 m/s, representing high agreement with stereophotogrammetry. Critically, in the context of longitudinal monitoring, where DMOs are aggregated across multiple walking bouts (e.g., weekly aggregates), random error is expected to average out, leaving bias as the more representative indicator of accuracy for detecting change over time; here, the pipeline demonstrated a near-zero walking speed bias (−0.01 m/s), well within the 0.10–0.20 m/s range considered clinically meaningful for gait speed change across patient groups [34], supporting its potential for detecting real, clinically important change in pre-post or longitudinal deployment. A Bland–Altman analysis revealed that cadence errors were well distributed with no systematic bias (Figure 2). Stride length errors fell within acceptable limits of agreement, though the Bland–Altman plot revealed a clear negative proportional bias, with the pipeline overestimating at shorter stride lengths and underestimating at longer stride lengths, consistent with the speed-dependent nature of the intensity-based Weinberg algorithm (Figure 3). Walking speed showed a similar pattern, driven by the stride length component (Figure 4).

### 4.2. Novelty

This is the first study to derive and validate composite pipeline blocks, including cadence, stride length, and walking speed, from a wrist-worn device, closing the gap between wrist accessibility and clinical validity. Previous work validated algorithms in isolation using reference-fed inputs [9]; the present study advances this to a full-pipeline evaluation where wearable-derived inputs are used at every stage. A novel scaled pipeline evaluation approach ensures that error propagation is realistically captured, and a novel walking speed performance index incorporating ICC, MAE, MARE, and limits of agreement extends the previous ranking frameworks [6,35] to whole-pipeline ranking.

### 4.3. Comparison with Previous Work in MLTCs

The per-participant analysis demonstrated that the population-best pipeline performed near-optimally for the majority of participants, with performance loss approaching zero for most individuals. Descriptively, participants with conditions confined to a single multimorbidity cluster showed higher mean loss across all DMOs compared with those spanning two or more clusters, suggesting that the pipeline is best suited to individuals with more complex multimorbidity profiles, consistent with the intended target population for which it was developed. The present sample was specifically recruited for this validation purpose and represents a real-world population of older adults with MLTC (age range: 65 to 90 years), characterised by at least one multimorbidity cluster according to UK Biobank categories for this age range [16]. This cohort was more clinically impaired than that in our previous validation study [9], which included younger participants with a primary condition and at least one comorbidity. Consequently, algorithmic ranking and validity differed between the two studies, supporting the notion that algorithm optimisation is population-specific, and that this more clinically impaired population requires a distinct algorithmic approach. Formal subsample analyses were not pursued as stratification would reduce walking bout counts below the pre-specified minimum of 100 required for adequate statistical power in the true-positive evaluation.

In terms of the ICD analysis, although Zijlstra IC ranked first at the population level, it was the individual-level best performer for only one participant. This apparent discrepancy reflects the narrow performance range across all ICD algorithms (PI range: 0.79–0.82) rather than true algorithmic superiority. Zijlstra IC demonstrated consistent, stable performance across all participants (albeit ranking first only in one participant), while other algorithms showed greater individual-level variability, despite ranking first in several participants. This stability justifies its selection as the universal ICD component.

### 4.4. Comparison with Mobilise-D Lower-Back Pipeline Validation

Pipeline performance was speed-dependent and task-complexity-dependent, consistent with Kirk et al. [6]. Our wrist-derived walking speed results (MAE: 0.13 m/s) are on par with those reported for lower-back sensors across multiple conditions (MAE: 0.10–0.14 m/s, population average: 0.12 m/s), which is expected given the inherently noisier wrist signal and the higher multimorbidity burden of the present sample. Cadence estimation showed the largest deviation from the validation work in Mobilise-D, with a MARE of approximately 10%, compared to 4.1% reported by Kirk et al. [6] for lower-back sensors. This is consistent with the aforementioned challenge of detecting step timing from the wrist, where signal complexity and arm swing variability make precise initial contact detection inherently more difficult than from a lumbar-mounted device. Stride length estimation from the wrist achieved a bias of −0.01 m, with limits of agreement of −0.31 to 0.29 m, and an absolute error of 0.14 m; this is marginally better than the lower-back Mobilise-D pipeline (bias: 0.04 m, LoA: −0.27 to 0.34 m, absolute error: 0.12 m) [6]. It should be noted that the Mobilise-D pipeline uses biomechanics-based SL methods with direct vertical displacement signals from a lumbar sensor, while the present pipeline relies solely on intensity-based adaptive algorithms developed for the wrist. Achieving near-zero bias at the population level from a sensor with no direct biomechanical grounding for spatial estimation demonstrates that appropriately fine-tuned wrist-adapted methods can match lower-back accuracy in laboratory settings, with significant implications for the clinical adoption of accessible, unobtrusive wearable technology.

### 4.5. Comparison with Best-Performing Wrist Algorithms

ElderNet is, to our knowledge, the only existing work beyond the present study to extract composite DMOs, including walking speed, cadence, and stride length, from a wrist-worn device [36]. It is a self-supervised deep learning model fine-tuned on the Mobilise-D TVS dataset. However, a critical methodological distinction applies to all ElderNet comparisons: validation was performed using reference-identified walking bouts of at least 10 s duration, rather than a fully wearable end-to-end pipeline. In the present multimorbid sample, the large majority of walking bouts are shorter than 10 s (Figure 4), meaning ElderNet’s validation conditions do not reflect the real-world deployment scenario faced here. For walking speed, ElderNet reported an MAE of 0.088 m/s. The present pipeline reports an overall MAE of 0.13 m/s; however, when restricted to bouts exceeding 10 s (equivalent validation conditions), walking speed absolute error matches or exceeds (60–120 s WBs, Figure 4) ElderNet’s accuracy despite operating on a fully wearable cascade in a considerably more impaired population. For cadence, ElderNet achieved an MAE of 3.11 steps/min, outperforming the present pipeline and highlighting initial contact detection as the primary area where deep learning offers a clear advantage over traditional wrist-adapted signal processing. For stride length, ElderNet reported an MAE of 11.61 cm compared to 14 cm in the present study; however, this difference is further confounded by the healthier Mobilise-D TVS cohort. Notably, stride length error in the present pipeline drops and stabilises above the 10 s bout duration threshold (Supplementary Figure S2), suggesting that the overall error is disproportionately driven by very short bouts. Both approaches demonstrate that wrist-derived composite DMO estimation is now approaching clinical utility. However, this comparison should be interpreted cautiously, as ElderNet, a self-supervised learning foundation model with supervised deep learning training on free-living data, may have learned more generalisable gait features than those captured by the rule-based signal processing methods used in the present manuscript, which may not transfer as well to real-world deployment.

### 4.6. Limitations, Future Research Directions, and Contextualisation Within Prior Single-Condition Cohorts

The present validation was conducted under laboratory conditions, where most walking bouts were very short, inflating overall error estimates. A sensitivity analysis across TP overlap thresholds (0.50–0.95) showed no meaningful accuracy–coverage trade-off, with performance indices remaining stable despite substantially fewer retained walking bouts up to 0.85; above 0.90, the performance indices became unstable and, in some cases, improved, but this improvement cannot be claimed due to the underpowered sample (less than 100 WBs) at these thresholds (Supplementary Figure S5). The duration distribution of walking bouts excluded from the TP evaluation resembled that of the included bouts, with the majority of both being shorter than 10 s. Nonetheless, bouts exceeding 60 s represented a smaller proportion of excluded bouts, reflecting improved detection performance between the wearable and reference systems for longer walking bouts compared to small sparse walking bouts (Supplementary Figure S6). However, this reflects the genuine mobility capacity of the target population, who have high multimorbidity burden and severely impaired gait, for whom prolonged instrumented assessments in real-world conditions are not clinically feasible. Indeed, comfortable walking speed in the present cohort was slower than in most previously validated Mobilise-D single-condition cohorts (CHF, COPD, MS, PD), with the exception of PFF [6], reflecting the compounded gait impairment associated with multimorbidity rather than a single disease process. Additionally, wrist movement patterns in a controlled laboratory setting may not fully reflect the complexity and variability of free-living arm swing, which could affect pipeline performance in real-world deployment [3,37]. The present study, alongside the latest work by our team and Mobilise-D [6,9], may represent the ceiling of signal processing-based approaches for gait estimation. All three DMOs showed regression slopes below unity (CAD: 0.52; SL: 0.30; WS: 0.39), indicating that wearable-derived estimates do not track the full dynamic range of the reference system, with the most pronounced compression in stride length and walking speed. This is most likely due to the limited nature of the rule-based/signal-processing algorithms. This compression is partly reflected by the narrower SD values from the wearable-derived DMOs in Table 7. While population-level validity is encouraging, the observed limits of agreement and moderate ICC values for stride length and walking speed suggest that individual-level clinical deployment, which requires detecting meaningful change within a single patient, warrants further development and validation. The logical next step is the development of robust deep learning and transfer learning models designed to operate within the newly developed modular pipeline to further improve accuracy while being capable of covering the full range of real-world gait-analysis needs.

## 5. Conclusions

This study presents the first validated wrist-worn modular pipeline for the estimation of cadence, stride length, and walking speed in older adults with multiple long-term conditions. The pipeline achieves walking speed accuracy comparable to lower-back sensors and, for bouts exceeding 10 s, is comparable to deep learning approaches, despite being evaluated in a more clinically impaired population using a fully wearable cascade. Cadence estimation remains the primary bottleneck, reflecting the inherent challenge of initial contact detection from the wrist. The modular design, in which algorithms are independently optimised for each DMO, demonstrated robustness across a clinically heterogeneous sample, with the population-best pipeline performing near-optimally for the majority of participants. These findings support the clinical adoption of wrist-worn devices for objective gait assessment in multimorbid populations and establish a validated open-source pipeline as a foundation for real-world deployment and future personalised approaches to care.

## Figures and Tables

**Table 1:** Sociodemographic and clinical characteristics of 45 people with MLTC.

Variable	Value
Participants, n	45
Age (years)	75.2 (6.5)
Height (cm)	164.6 (13.3)
Weight (kg)	86.9 (18.0)
BMI (kg/m2)	33.0 (11.8)
Male sex, n (%)	17 (37.0%)
4-MGS (s)	5.01 (1.38)
5× Sit-to-Stand time (s)	18.7 (6.4)
VAS pain while walking (0–100)	34.9 (26.3)
VAS resting pain (0–100)	31.8 (25.0)
FCI total score	4 (0–11)
Number of medications	6 (0–13)
Fallers in past 12 months, n (%)	17 (37.7%)
Walking aid use outdoors, n (%)	12 (26.6%)
Multimorbidity clusters (non-mutually exclusive), n (%)	
- Cardiometabolic	42 (93.3%)
- Painful conditions	41 (91.1%)
- Pulmonary	18 (40.0%)
- Cancer	11 (24.4%)
Number of clusters per participant	2 (1–4)
Number of clusters per participant, n (%)	
- 1 cluster	4 (8.8%)
- 2 clusters	21 (46.6%)
- 3 clusters	14 (31.1%)
- 4 clusters	6 (13.3%)

BMI: body mass index; 4-MGS: four-metre gait speed; VAS: visual analogue scale; FCI: Functional Comorbidity Index. Values are presented as mean (SD), n (%), or median (range), as appropriate.

**Table 2:** Gait sequence detection algorithm performance metrics ranked by performance index, with all metrics reported as mean values with 95% confidence intervals [95% CI]; algorithm versions are wrist-optimised adaptations from [9].

Algorithm	Mean Performance Index	n WBs	ICC	Specificity	Accuracy	Recall	Precision
Ionescu [18]	0.72	500	0.62 [0.51, 0.71]	0.80 [0.77, 0.83]	0.85 [0.82, 0.87]	0.90 [0.84, 0.96]	0.74 [0.68, 0.79]
Iluz [19]	0.69	650	0.77 [0.62, 0.86]	0.72 [0.65, 0.80]	0.84 [0.80, 0.88]	0.89 [0.83, 0.95]	0.86 [0.80, 0.91]
Ionescu * [18]	0.68	469	0.61 [0.51, 0.70]	0.88 [0.86, 0.91]	0.83 [0.80, 0.86]	0.78 [0.71, 0.85]	0.78 [0.71, 0.84]
Keren * [20]	0.68	645	0.55 [0.45, 0.65]	0.90 [0.87, 0.92]	0.79 [0.76, 0.83]	0.69 [0.60, 0.77]	0.74 [0.66, 0.81]
Keren [20]	0.68	646	0.55 [0.45, 0.65]	0.90 [0.87, 0.92]	0.79 [0.76, 0.83]	0.69 [0.60, 0.77]	0.73 [0.66, 0.81]
MacLean [21]	0.67	514	0.58 [0.45, 0.70]	0.80 [0.74, 0.85]	0.81 [0.73, 0.89]	0.81 [0.72, 0.91]	0.92 [0.86, 0.99]
Hickey [22]	0.6	447	0.46 [0.36, 0.56]	0.91 [0.87, 0.94]	0.80 [0.75, 0.84]	0.69 [0.61, 0.78]	0.70 [0.61, 0.78]
Kheirkhahan [23]	0.6	380	0.52 [0.41, 0.64]	0.67 [0.58, 0.76]	0.73 [0.65, 0.82]	0.73 [0.63, 0.84]	0.86 [0.78, 0.95]

* Adaptive version. ICC: intraclass correlation coefficient; n WBs: number of detected gait sequences. Variation in n WBs across algorithms reflects differences in wearable gait sequence detection outputs.

**Table 3:** Initial contact detection algorithm performance metrics evaluated within the wearable pipeline utilising the Ionescu GSD algorithm, ranked by performance index, with all metrics reported as mean values with 95% confidence intervals [95% CI]; algorithm versions are wrist-optimised adaptations from [9].

Algorithm	Mean Performance Index	n WBs	Recall	Precision	Absolute Timing Error (s)	Relative Timing Error (%)
Zijlstra [24]	0.82	145	0.76 [0.66, 0.85]	0.82 [0.72, 0.92]	0.13 [0.10, 0.17]	0.22 [0.16, 0.27]
McCamley [25]	0.82	145	0.77 [0.67, 0.88]	0.78 [0.69, 0.86]	0.12 [0.10, 0.15]	0.21 [0.15, 0.25]
Ducharme [26]	0.81	145	0.77 [0.67, 0.88]	0.79 [0.70, 0.89]	0.13 [0.10, 0.17]	0.21 [0.16, 0.27]
Gu * [27]	0.81	145	0.75 [0.66, 0.84]	0.78 [0.69, 0.87]	0.13 [0.10, 0.16]	0.21 [0.16, 0.26]
Gu [27]	0.81	145	0.74 [0.66, 0.84]	0.78 [0.70, 0.86]	0.13 [0.10, 0.16]	0.21 [0.16, 0.26]
Micó-Amigo [28]	0.81	134	0.81 [0.69, 0.93]	0.69 [0.59, 0.78]	0.11 [0.09, 0.14]	0.18 [0.14, 0.22]
HKLee [29]	0.8	145	0.72 [0.60, 0.84]	0.80 [0.69, 0.91]	0.13 [0.10, 0.17]	0.22 [0.17, 0.27]
Pham [30]	0.8	145	0.73 [0.61, 0.85]	0.77 [0.66, 0.89]	0.13 [0.10, 0.17]	0.21 [0.16, 0.27]
Shin [31]	0.79	142	0.67 [0.53, 0.81]	0.78 [0.64, 0.92]	0.13 [0.10, 0.16]	0.20 [0.15, 0.26]

* Adaptive version; n WBs: number of true-positive walking bouts. Minor variation in n WBs across algorithms reflects cases where one algorithm detected no initial contacts within a walking bout, resulting in exclusion from downstream metric computation.

**Table 4:** Cadence pipeline performance metrics for each ICD algorithm combined with Ionescu GSD, ranked by performance index, with all metrics reported as mean values with 95% confidence intervals [95% CI]; algorithm versions are wrist-optimised adaptations from [9].

ICD Method	Mean Performance Index	N WBs	Absolute Error	Relative Error (%)	ICC	Bias [LoA] (Steps/min)	Wearable CAD (Steps/Min)	Reference CAD (Steps/Min)
Zijlstra [24]	0.78	145	5.71 [2.16, 9.22]	5.75 [2.43, 9.00]	0.70 [0.50, 0.86]	0.98 [−16.45, 17.02]	99.00 [94.83, 103.36]	98.02 [94.14, 103.48]
Ducharme [26]	0.76	145	6.29 [2.47, 11.28]	6.25 [2.86, 11.14]	0.65 [0.45, 0.83]	−0.04 [−19.76, 20.31]	97.99 [94.20, 103.96]	98.02 [94.14, 103.48]
Gu * [27]	0.75	145	7.47 [2.83, 12.77]	7.50 [2.86, 12.43]	0.62 [0.42, 0.81]	0.10 [−23.89, 22.63]	98.12 [93.10, 103.26]	98.02 [94.14, 103.48]
Gu [27]	0.74	145	7.85 [3.06, 13.41]	7.75 [3.00, 12.86]	0.60 [0.40, 0.79]	−0.62 [−25.40, 22.51]	97.41 [91.90, 102.82]	98.02 [94.14, 103.48]
McCamleyc [25]	0.71	145	11.36 [4.04, 15.05]	11.25 [4.14, 14.43]	0.56 [0.36, 0.77]	0.78 [−29.77, 24.68]	98.81 [90.05, 102.48]	98.02 [94.14, 103.48]
Pham [30]	0.7	145	15.71 [−2.95, 38.08]	15.62 [−3.43, 38.14]	0.50 [0.28, 0.73]	4.15 [−74.91, 83.62]	102.17 [81.99, 124.34]	98.02 [94.14, 103.48]
HKLee [29]	0.68	145	10.33 [4.07, 19.32]	9.62 [4.14, 17.71]	0.49 [0.37, 0.68]	−8.68 [−40.52, 20.91]	89.35 [82.66, 95.34]	98.02 [94.14, 103.48]
Shin [31]	0.67	145	12.77 [5.89, 22.45]	12.00 [5.86, 20.29]	0.50 [0.41, 0.68]	−11.83 [−44.88, 18.68]	86.19 [79.14, 92.28]	98.02 [94.14, 103.48]
Micó-Amigo [28]	0.61	131	52.52 [21.26, 67.72]	54.62 [21.43, 70.00]	0.50 [0.30, 0.72]	38.68 [−78.14, 135.50]	136.31 [98.49, 155.57]	97.62 [93.31, 103.39]

ICC: intraclass correlation coefficient; CAD: cadence; LoA: limits of agreement; n WBs: number of true-positive walking bouts; 95% CI: 95% confidence interval. * Adaptive version.

**Table 5:** Stride length pipeline performance metrics for each ICD and SL algorithm combination with Ionescu GSD, ranked by performance index, with all metrics reported as mean values with 95% confidence intervals [95% CI]; algorithm versions are wrist-optimised adaptations from [9].

SL Method	ICD Method	Mean Performance Index	N WBs	Absolute Error	Relative Error (%)	ICC	Bias [LoA] (m)	Detected Stride Length (m)	Reference Stride Length (m)
Weinberg * [32]	Pham [30]	0.78	145	0.14 [0.07, 0.18]	14.50 [6.71, 21.14]	0.64 [0.44, 0.82]	−0.05 [−0.29, 0.25]	0.96 [0.91, 0.99]	1.01 [0.89, 1.04]
Weinberg * [32]	Zijlstra [24]	0.78	145	0.14 [0.08, 0.18]	14.62 [7.14, 20.57]	0.65 [0.44, 0.82]	−0.05 [−0.28, 0.24]	0.96 [0.91, 0.99]	1.01 [0.89, 1.04]
Weinberg * [32]	Gu [27]	0.78	145	0.15 [0.08, 0.17]	14.88 [7.71, 19.86]	0.66 [0.45, 0.83]	−0.07 [−0.29, 0.22]	0.94 [0.90, 0.97]	1.01 [0.89, 1.04]
Weinberg * [32]	Ducharme [26]	0.78	145	0.14 [0.08, 0.17]	14.50 [7.14, 20.14]	0.66 [0.46, 0.83]	−0.06 [−0.28, 0.23]	0.95 [0.91, 0.98]	1.01 [0.89, 1.04]
Weinberg [32]	Gu [27]	0.77	145	0.16 [0.09, 0.18]	15.50 [8.43, 19.57]	0.64 [0.44, 0.82]	−0.10 [−0.32, 0.18]	0.91 [0.87, 0.93]	1.01 [0.89, 1.04]
Weinberg * [32]	Shin [31]	0.77	145	0.15 [0.09, 0.18]	15.25 [7.71, 20.86]	0.63 [0.44, 0.81]	−0.07 [−0.31, 0.22]	0.93 [0.89, 0.97]	1.01 [0.89, 1.04]
Weinberg * [32]	McCamley [25]	0.77	145	0.15 [0.08, 0.18]	15.38 [6.86, 21.00]	0.64 [0.44, 0.82]	−0.06 [−0.29, 0.24]	0.95 [0.91, 0.99]	1.01 [0.89, 1.04]
Weinberg * [32]	HKLee [29]	0.77	145	0.15 [0.08, 0.18]	15.12 [7.14, 21.29]	0.63 [0.44, 0.81]	−0.05 [−0.29, 0.25]	0.96 [0.92, 0.99]	1.01 [0.89, 1.04]
Weinberg * [32]	Gu * [27]	0.77	145	0.15 [0.08, 0.18]	15.12 [7.57, 20.14]	0.64 [0.45, 0.82]	−0.07 [−0.29, 0.22]	0.94 [0.90, 0.97]	1.01 [0.89, 1.04]
Weinberg [32]	Zijlstra [24]	0.77	145	0.15 [0.09, 0.18]	14.88 [7.86, 19.57]	0.64 [0.44, 0.82]	−0.09 [−0.31, 0.19]	0.92 [0.88, 0.94]	1.01 [0.89, 1.04]
Weinberg [32]	Pham [30]	0.77	145	0.15 [0.09, 0.18]	14.75 [7.71, 19.86]	0.64 [0.43, 0.81]	−0.09 [−0.32, 0.20]	0.92 [0.88, 0.94]	1.01 [0.89, 1.04]
Weinberg [32]	McCamley [25]	0.77	145	0.16 [0.09, 0.18]	15.75 [8.14, 20.29]	0.63 [0.43, 0.80]	−0.09 [−0.32, 0.20]	0.92 [0.88, 0.94]	1.01 [0.89, 1.04]
Weinberg [32]	Ducharme [26]	0.77	145	0.16 [0.09, 0.18]	15.38 [8.43, 19.43]	0.64 [0.44, 0.82]	−0.09 [−0.31, 0.19]	0.91 [0.88, 0.94]	1.01 [0.89, 1.04]
Weinberg [32]	HKLee [29]	0.76	145	0.15 [0.09, 0.18]	15.25 [8.43, 20.14]	0.62 [0.43, 0.80]	−0.08 [−0.31, 0.21]	0.92 [0.89, 0.95]	1.01 [0.89, 1.04]
Weinberg [32]	Gu * [27]	0.76	145	0.16 [0.09, 0.18]	15.75 [8.57, 19.86]	0.63 [0.43, 0.81]	−0.10 [−0.32, 0.18]	0.91 [0.88, 0.92]	1.01 [0.89, 1.04]
Weinberg [32]	Shin [31]	0.76	145	0.17 [0.09, 0.19]	15.75 [9.00, 20.43]	0.62 [0.42, 0.80]	−0.11 [−0.33, 0.18]	0.90 [0.86, 0.92]	1.01 [0.89, 1.04]
Weinberg * [32]	Micó-Amigo [28]	0.76	131	0.17 [0.08, 0.20]	16.88 [7.43, 22.29]	0.62 [0.42, 0.81]	−0.10 [−0.34, 0.22]	0.91 [0.86, 0.96]	1.01 [0.89, 1.05]
Weinberg [32]	Micó-Amigo [28]	0.75	131	0.18 [0.10, 0.20]	17.25 [8.71, 21.71]	0.61 [0.41, 0.79]	−0.13 [−0.36, 0.18]	0.88 [0.84, 0.92]	1.01 [0.89, 1.05]
Bylemans *^ [33]	Shin [31]	0.73	145	0.20 [0.10, 0.23]	19.25 [9.29, 25.86]	0.58 [0.39, 0.77]	−0.13 [−0.44, 0.26]	0.88 [0.81, 0.95]	1.01 [0.89, 1.04]
Bylemans *^ [33]	McCamley [25]	0.73	145	0.20 [0.10, 0.23]	19.62 [9.29, 26.14]	0.58 [0.37, 0.77]	−0.07 [−0.41, 0.36]	0.94 [0.87, 1.02]	1.01 [0.89, 1.04]
Bylemans *^ [33]	HKLee [29]	0.73	145	0.19 [0.10, 0.23]	19.25 [9.71, 25.14]	0.58 [0.39, 0.78]	−0.08 [−0.40, 0.32]	0.92 [0.86, 1.00]	1.01 [0.89, 1.04]
Bylemans *^ [33]	Gu * [27]	0.72	145	0.19 [0.11, 0.23]	18.63 [10.29, 24.43]	0.56 [0.36, 0.76]	−0.13 [−0.43, 0.22]	0.87 [0.81, 0.92]	1.01 [0.89, 1.04]
Bylemans *^ [33]	Pham [30]	0.72	145	0.21 [0.10, 0.25]	21.12 [9.00, 28.86]	0.56 [0.36, 0.76]	−0.08 [−0.44, 0.38]	0.92 [0.85, 1.03]	1.01 [0.89, 1.04]
Bylemans *^ [33]	Zijlstra [24]	0.71	145	0.20 [0.10, 0.24]	19.62 [8.43, 28.00]	0.53 [0.33, 0.74]	−0.07 [−0.43, 0.38]	0.94 [0.86, 1.02]	1.01 [0.89, 1.04]
Bylemans *^ [33]	Micó-Amigo [28]	0.71	131	0.20 [0.11, 0.28]	20.38 [10.71, 31.00]	0.56 [0.35, 0.76]	−0.00 [−0.45, 0.51]	1.01 [0.90, 1.10]	1.01 [0.89, 1.05]
Bylemans *^ [33]	Gu * [27]	0.71	145	0.19 [0.11, 0.23]	18.75 [10.14, 25.00]	0.55 [0.37, 0.75]	−0.14 [−0.44, 0.21]	0.86 [0.80, 0.91]	1.01 [0.89, 1.04]
Bylemans *^ [33]	Ducharme [26]	0.71	145	0.19 [0.09, 0.24]	19.50 [8.00, 28.14]	0.53 [0.33, 0.75]	−0.07 [−0.42, 0.37]	0.94 [0.87, 1.02]	1.01 [0.89, 1.04]

SL: stride length; ICC: intraclass correlation coefficient; LoA: limits of agreement; 95% CI: 95% confidence interval. * Adaptive version; ^ foot-length-augmented version. n WBs: number of true-positive walking bouts; minor variation in n WBs across algorithms reflects cases where one algorithm detected no initial contacts within a walking bout, resulting in exclusion from downstream metric computation.

**Table 6:** Walking speed pipeline performance metrics for each ICD and SL algorithm combination with Ionescu GSD, ranked by performance index, with all metrics reported as mean values with 95% confidence intervals [95% CI]; algorithm versions are wrist-optimised adaptations from [9].

CAD Method	SL Method	Mean Performance Index	N WBs	Absolute Error	Relative Error (%)	ICC	Bias [LoA] (m/s)	Wearable WS (m/s)	Reference WS (m/s)
Zijlstra [24]	Weinberg * [32]	0.78	145	0.12 [0.07, 0.16]	14.62 [8.43, 21.00]	0.68 [0.45, 0.84]	−0.04 [−0.27, 0.22]	0.79 [0.73, 0.83]	0.82 [0.73, 0.87]
Ducharme [26]	Weinberg * [32]	0.77	145	0.14 [0.07, 0.17]	16.50 [8.57, 22.00]	0.65 [0.43, 0.83]	−0.05 [−0.28, 0.24]	0.77 [0.73, 0.83]	0.82 [0.73, 0.87]
Zijlstra [24]	Weinberg [32]	0.77	145	0.12 [0.07, 0.16]	14.88 [8.71, 20.86]	0.66 [0.44, 0.82]	−0.07 [−0.29, 0.19]	0.76 [0.71, 0.79]	0.82 [0.73, 0.87]
McCamley [25]	Weinberg * [32]	0.77	145	0.11 [0.07, 0.16]	14.25 [8.71, 21.57]	0.67 [0.45, 0.84]	−0.05 [−0.30, 0.21]	0.77 [0.70, 0.81]	0.82 [0.73, 0.87]
Gu * [27]	Weinberg * [32]	0.77	145	0.13 [0.07, 0.16]	15.75 [8.29, 21.71]	0.64 [0.43, 0.82]	−0.06 [−0.31, 0.22]	0.77 [0.72, 0.81]	0.82 [0.73, 0.87]
Ducharme [26]	Weinberg [32]	0.76	145	0.14 [0.08, 0.16]	16.50 [8.71, 21.29]	0.64 [0.43, 0.81]	−0.08 [−0.31, 0.20]	0.74 [0.70, 0.79]	0.82 [0.73, 0.87]
McCamley [25]	Weinberg [32]	0.76	145	0.12 [0.08, 0.17]	15.12 [9.71, 21.71]	0.65 [0.44, 0.82]	−0.08 [−0.33, 0.18]	0.75 [0.68, 0.78]	0.82 [0.73, 0.87]
Gu * [27]	Weinberg [32]	0.76	145	0.13 [0.08, 0.17]	16.25 [8.71, 21.57]	0.62 [0.40, 0.79]	−0.09 [−0.33, 0.19]	0.74 [0.69, 0.77]	0.82 [0.73, 0.87]
Gu [27]	Weinberg * [32]	0.75	145	0.13 [0.07, 0.17]	16.38 [8.43, 23.29]	0.60 [0.40, 0.79]	−0.07 [−0.32, 0.22]	0.76 [0.71, 0.80]	0.82 [0.73, 0.87]
Gu [27]	Weinberg [32]	0.74	145	0.14 [0.08, 0.18]	17.25 [9.57, 23.57]	0.58 [0.37, 0.77]	−0.09 [−0.35, 0.20]	0.73 [0.69, 0.77]	0.82 [0.73, 0.87]
HKLee [29]	Weinberg * [32]	0.74	145	0.15 [0.09, 0.20]	17.88 [11.14, 23.86]	0.61 [0.42, 0.78]	−0.11 [−0.38, 0.19]	0.71 [0.65, 0.76]	0.82 [0.73, 0.87]
Gu * [27]	Bylemans *^ [33]	0.73	145	0.16 [0.10, 0.20]	19.00 [11.14, 25.29]	0.60 [0.38, 0.78]	−0.11 [−0.39, 0.21]	0.72 [0.65, 0.77]	0.82 [0.73, 0.87]
HKLee [29]	Weinberg [32]	0.73	145	0.17 [0.10, 0.21]	19.25 [12.43, 25.00]	0.59 [0.41, 0.76]	−0.14 [−0.41, 0.16]	0.69 [0.63, 0.73]	0.82 [0.73, 0.87]
McCamley [25]	Bylemans *^ [33]	0.73	145	0.14 [0.08, 0.20]	17.25 [10.14, 25.86]	0.65 [0.43, 0.82]	−0.05 [−0.38, 0.31]	0.78 [0.68, 0.85]	0.82 [0.73, 0.87]
Zijlstra [24]	Bylemans *^ [33]	0.73	145	0.15 [0.08, 0.21]	19.12 [8.57, 27.71]	0.61 [0.38, 0.80]	−0.05 [−0.37, 0.34]	0.78 [0.71, 0.86]	0.82 [0.73, 0.87]
Shin [31]	Weinberg * [32]	0.73	145	0.18 [0.11, 0.23]	20.38 [13.71, 26.14]	0.60 [0.43, 0.76]	−0.16 [−0.42, 0.13]	0.67 [0.60, 0.71]	0.82 [0.73, 0.87]
Pham [30]	Weinberg [32]	0.72	145	0.15 [0.08, 0.22]	18.50 [9.43, 27.14]	0.59 [0.38, 0.77]	−0.08 [−0.45, 0.32]	0.74 [0.67, 0.81]	0.82 [0.73, 0.87]
Shin [31]	Weinberg [32]	0.72	145	0.20 [0.13, 0.25]	22.38 [15.43, 28.14]	0.58 [0.43, 0.74]	−0.18 [−0.45, 0.11]	0.64 [0.59, 0.68]	0.82 [0.73, 0.87]
Pham [30]	Weinberg * [32]	0.72	145	0.15 [0.08, 0.21]	17.88 [9.00, 27.86]	0.60 [0.38, 0.79]	−0.05 [−0.42, 0.36]	0.78 [0.69, 0.85]	0.82 [0.73, 0.87]
Ducharme [26]	Bylemans *^ [33]	0.72	145	0.17 [0.08, 0.21]	20.88 [9.14, 29.43]	0.59 [0.37, 0.79]	−0.05 [−0.38, 0.35]	0.78 [0.71, 0.87]	0.82 [0.73, 0.87]
HKLee [29]	Bylemans *^ [33]	0.71	145	0.18 [0.10, 0.23]	21.75 [12.71, 28.14]	0.59 [0.38, 0.78]	−0.13 [−0.46, 0.24]	0.69 [0.61, 0.77]	0.82 [0.73, 0.87]
Gu [27]	Bylemans *^ [33]	0.7	145	0.17 [0.10, 0.22]	20.38 [11.43, 28.86]	0.54 [0.35, 0.74]	−0.12 [−0.43, 0.23]	0.70 [0.63, 0.76]	0.82 [0.73, 0.87]
Shin [31]	Bylemans *^ [33]	0.69	145	0.21 [0.12, 0.27]	24.88 [14.86, 32.00]	0.58 [0.41, 0.75]	−0.19 [−0.51, 0.17]	0.64 [0.56, 0.71]	0.82 [0.73, 0.87]
Pham [30]	Bylemans *^ [33]	0.64	145	0.26 [0.03, 0.47]	33.88 [−1.29, 68.71]	0.55 [0.34, 0.76]	−0.01 [−0.85, 0.93]	0.82 [0.60, 1.08]	0.82 [0.73, 0.87]
Micó-Amigo [28]	Weinberg [32]	0.6	131	0.32 [0.16, 0.42]	39.12 [20.00, 55.43]	0.55 [0.34, 0.76]	0.16 [−0.57, 0.79]	0.98 [0.73, 1.09]	0.82 [0.73, 0.87]
Micó-Amigo [28]	Weinberg * [32]	0.59	131	0.33 [0.17, 0.44]	41.25 [21.00, 58.00]	0.56 [0.35, 0.77]	0.19 [−0.56, 0.84]	1.01 [0.76, 1.13]	0.82 [0.73, 0.87]
Micó-Amigo [28]	Bylemans *^ [33]	0.52	131	0.54 [0.19, 0.84]	66.50 [23.86, 105.14]	0.54 [0.34, 0.74]	0.39 [−1.18, 1.85]	1.21 [0.74, 1.53]	0.82 [0.73, 0.87]

WS: walking speed; ICC: intraclass correlation coefficient; LoA: limits of agreement; N WBs: number of true-positive walking bouts; 95% CI: 95% confidence interval. * Adaptive version; ^ foot-length-augmented version.

**Table 7:** Combined evaluation performance metrics for the best-performing pipeline (Ionescu GSD + Zijlstra/Pham IC + Weinberg SL adaptive) across stride length, cadence, and walking speed estimation, with all metrics reported as mean values with 95% confidence intervals [95% CI].

DMO	N WBs	Absolute Error	Relative Error (%)	ICC	Bias [LoA]	Detected	Reference
Cadence (steps/min)	504	7.71 [5.17, 10.26]	8.38 [5.38, 11.12]	0.70 [0.56, 0.81]	1.42 [−18.68, 21.52]	97.83 (11.91)	96.41 (13.22)
Stride length (m)	504	0.14 [0.11, 0.17]	15.38 [11.12, 20.00]	0.66 [0.51, 0.78]	−0.01 [−0.31, 0.29]	0.95 (0.11)	0.95 (0.23)
Walking speed (m/s)	504	0.13 [0.10, 0.16]	17.88 [13.00, 22.12]	0.67 [0.52, 0.79]	−0.01 [−0.28, 0.25]	0.77 (0.14)	0.78 (0.25)

N WBs: total wearable-detected walking bouts; ICC: intraclass correlation coefficient; LoA: limits of agreement; 95% CI: 95% confidence interval. Combined evaluation includes all wearable-detected walking bouts regardless of reference system overlap. Detected and reference values are presented as mean (SD).

## Data Availability

At the time of writing, the data presented in this study are not publicly available due to institutional and ethical restrictions, but may be made available in the future, subject to institutional approval.

## Supplementary Materials

The following supporting information can be downloaded at: https://www.mdpi.com/article/10.3390/s26154946/s1, Figure S1: Overview of the data acquisition and processing pipeline, showing the two synchronised data streams (wrist-worn IMU and stereophotogrammetry) recorded during laboratory tasks, their independent processing into wearable and reference digital mobility outcomes (DMOs), and the subsequent performance assessment; Figure S2: Stride length relative error versus walking bout duration for the best-performing pipeline; Figure S3: Stride length relative error versus reference walking speed for the best-performing pipeline; Figure S4: Absolute walking speed error by laboratory task for the best-performing pipeline; Figure S5: Mean performance index for cadence (CAD), stride length (SL), and walking speed (WS) as a function of the GSD overlap threshold used to define true positive walking bouts, using the finalised algorithms from the modular population-best pipeline. Grey bars show the number of walking bouts retained at each threshold (n WB, right axis); Figure S6: Distribution of walking bout durations for wearable-detected bouts excluded from the true positive (TP) evaluation (overlap ≤85% with the reference system); Figure S7: Scatter plots of wearable-derived versus reference-derived (A) cadence, (B) stride length, and (C) walking speed values across all laboratory tasks (complete-case analysis; nWB = 504). Dashed line indicates identity (y = x); solid line indicates the fitted linear regression, with slope reported for each panel; Table S1: GSD performance index weights; Table S2: ICD performance index weights; Table S3: SL performance index weights; Table S4: CAD performance index weights; Table S5: WS performance index weights; Table S6: Stride length algorithm performance metrics evaluated using reference-derived walking bouts and initial contacts; Table S7: Mean and median percentage performance loss from the population-best modular pipeline by number of multimorbidity clusters.

## Abbreviations

The following abbreviations are used in this manuscript:

DMO	Digital mobility outcome
MLTC	Multiple long-term conditions
GSD	Gait sequence detection
ICD	Initial contact detection
IC	Initial contact
SL	Stride length
CAD	Cadence
WS	Walking speed
WB	Walking bout
IMU	Inertial measurement unit
SP	Stereophotogrammetry
ICC	Intraclass correlation coefficient
CI	Confidence interval
MAE	Mean absolute error
MARE	Mean absolute relative error
LoA	Limits of agreement
PI	Performance index
RMSSD	Root mean square of successive differences
CV	Coefficient of variation
TUG	Timed Up and Go
CWT	Continuous Walking Test
SDA	Simulated Daily Activities
4-MGS	Four-metre gait speed
BMI	Body mass index
VAS	Visual analogue scale
FCI	Functional Comorbidity Index
TVS	Technical validation study
CHF	Chronic heart failure
COPD	Chronic obstructive pulmonary disease
MS	Multiple sclerosis
PD	Parkinson’s disease
PFF	Proximal femur fracture
SD	Standard deviation
NHS	National Health Service
IRAS	Integrated Research Application System

## References

[1] Megaritis D., Long M., Heras M.D.L., Alcaraz V., Alvarez P., Buekers J., Becker C., Braun J., Chynkiamis N., Delgado-Ortiz L. (2026). The Construct Validity of Real-World Digital Mobility Outcomes in People with Chronic Obstructive Pulmonary Disease. ERJ Open Res..

[2] Brittain G., Buckley E., Lanfranchi V., Long M., Tsaktanis T., Rothhammer V., Hansen C., Stürner K.H., Maetzler W., Rochester L. (2026). Recruitment of the multiple sclerosis cohort within the European Mobilise-D clinical validation study-lessons learnt, baseline demographics and clinical characteristics. Trials.

[3] Warmerdam E., Hausdorff J.M., Atrsaei A., Zhou Y., Mirelman A., Aminian K., Espay A.J., Hansen C., Evers L.J.W., Keller A. (2020). Long-term unsupervised mobility assessment in movement disorders. Lancet Neurol..

[4] Studenski S., Perera S., Patel K., Rosano C., Faulkner K., Inzitari M., Brach J., Chandler J., Cawthon P., Connor E.B. (2011). Gait speed and survival in older adults. JAMA.

[5] Bakker J.P., Barge R., Centra J., Cobb B., Cota C., Guo C.C., Hartog B., Horowicz-Mehler N., Izmailova E.S., Manyakov N.V. (2025). V3+ extends the V3 framework to ensure user-centricity and scalability of sensor-based digital health technologies. npj Digit. Med..

[6] Kirk C., Küderle A., Micó-Amigo M.E., Bonci T., Paraschiv-Ionescu A., Ullrich M., Soltani A., Gazit E., Salis F., Alcock L. (2024). Mobilise-D insights to estimate real-world walking speed in multiple conditions with a wearable device. Sci. Rep..

[7] Ryan A., Wallace E., O’hara P., Smith S.M. (2015). Multimorbidity and functional decline in community-dwelling adults: A systematic review. Health Qual. Life Outcomes.

[8] Barnett K., Mercer S.W., Norbury M., Watt G., Wyke S., Guthrie B. (2012). Epidemiology of multimorbidity and implications for health care, research, and medical education: A cross-sectional study. Lancet.

[9] Megaritis D., Alcock L., Scott K., Hiden H., Cereatti A., Vogiatzis I., Del Din S. (2025). Real-World Wrist-Derived Digital Mobility Outcomes in People with Multiple Long-Term Conditions: A Comparison of Algorithms. Bioengineering.

[10] Mazzà C., Alcock L., Aminian K., Becker C., Bertuletti S., Bonci T., Brown P., Brozgol M., Buckley E., Carsin A.-E. (2021). Technical validation of real-world monitoring of gait: A multicentric observational study. BMJ Open.

[11] Salis F., Bertuletti S., Bonci T., Caruso M., Scott K., Alcock L., Buckley E., Gazit E., Hansen C., Schwickert L. (2023). A multi-sensor wearable system for the assessment of diseased gait in real-world conditions. Front. Bioeng. Biotechnol..

[12] Micó-Amigo M.E., Bonci T., Paraschiv-Ionescu A., Ullrich M., Kirk C., Soltani A., Küderle A., Gazit E., Salis F., Alcock L. (2023). Assessing real-world gait with digital technology? Validation, insights and recommendations from the Mobilise-D consortium. J. Neuroeng. Rehabil..

[13] Megaritis D. (2025). MultiGait: Real-World Gait Pipeline for Wrist-Worn Devices.

[14] Küderle A., Tasca P., Bicer M., Kirk C., Megaritis D., Hinchliffe C., Stihi A., Muecke A., Babar Z., Kluge F. (2025). MobGap—The Mobilise-D Algorithm Toolbox.

[15] Bonett D.G. (2002). Sample size requirements for estimating intraclass correlations with desired precision. Stat. Med..

[16] Steell L., Krauth S.J., Ahmed S., Dibben G.O., McIntosh E., Hanlon P., Lewsey J., Nicholl B.I., McAllister D.A., Smith S.M. (2025). Multimorbidity clusters and their associations with health-related quality of life in two UK cohorts. BMC Med..

[17] Krauth S.J., Steell L., Ahmed S., McIntosh E., Dibben G.O., Hanlon P., Lewsey J., Nicholl B.I., McAllister D.A., Smith S.M. (2024). Association of latent class analysis-derived multimorbidity clusters with adverse health outcomes in patients with multiple long-term conditions: Comparative results across three UK cohorts. EClinicalMedicine.

[18] Paraschiv-Ionescu A., Newman C.J., Carcreff L., Gerber C.N., Armand S., Aminian K. (2019). Locomotion and cadence detection using a single trunk-fixed accelerometer: Validity for children with cerebral palsy in daily life-like conditions. J. Neuroeng. Rehabil..

[19] Iluz T., Gazit E., Herman T., Sprecher E., Brozgol M., Giladi N., Mirelman A., Hausdorff J.M. (2014). Automated detection of missteps during community ambulation in patients with Parkinson’s disease: A new approach for quantifying fall risk in the community setting. J. Neuroeng. Rehabil..

[20] Keren K., Busse M., Fritz N.E., Muratori L.M., Gazit E., Hillel I., Scheinowitz M., Gurevich T., Inbar N., Omer N. (2021). Quantification of Daily-Living Gait Quantity and Quality Using a Wrist-Worn Accelerometer in Huntington’s Disease. Front. Neurol..

[21] MacLean M.K., Rehman R.Z.U., Kerse N., Taylor L., Rochester L., Del Din S. (2023). Walking Bout Detection for People Living in Long Residential Care: A Computationally Efficient Algorithm for a 3-Axis Accelerometer on the Lower Back. Sensors.

[22] Hickey A., Del Din S., Rochester L., Godfrey A. (2017). Detecting free-living steps and walking bouts: Validating an algorithm for macro gait analysis. Physiol. Meas..

[23] Kheirkhahan M., Chen Z., Corbett D.B., Wanigatunga A.A., Manini T.M., Ranka S. (2017). Adaptive walk detection algorithm using activity counts. 2017 IEEE EMBS International Conference on Biomedical & Health Informatics (BHI).

[24] Zijlstra W., Hof A.L. (2003). Assessment of spatio-temporal gait parameters from trunk accelerations during human walking. Gait Posture.

[25] McCamley J., Donati M., Grimpampi E., Mazzà C. (2012). An enhanced estimate of initial contact and final contact instants of time using lower trunk inertial sensor data. Gait Posture.

[26] Ducharme S.W., Lim J., Busa M.A., Aguiar E.J., Moore C.C., Schuna J.M., Barreira T.V., Staudenmayer J., Chipkin S.R., Tudor-Locke C. (2021). A Transparent Method for Step Detection using an Acceleration Threshold. J. Meas. Phys. Behav..

[27] Gu F., Khoshelham K., Shang J., Yu F., Wei Z. (2017). Robust and accurate smartphone-based step counting for indoor localization. IEEE Sens. J..

[28] Micó-Amigo M.E., Kingma I., Ainsworth E., Walgaard S., Niessen M., van Lummel R.C., van Dieën J.H. (2016). A novel accelerometry-based algorithm for the detection of step durations over short episodes of gait in healthy elderly. J. Neuroeng. Rehabil..

[29] Lee H.-K., You J., Cho S.-P., Hwang S.-J., Lee D.-R., Kim Y.-H., Lee K.-J. (2010). Computational methods to detect step events for normal and pathological gait evaluation using accelerometer. Electron. Lett..

[30] Pham M.H., Elshehabi M., Haertner L., Del Din S., Srulijes K., Heger T., Synofzik M., Hobert M.A., Faber G.S., Hansen C. (2017). Validation of a Step Detection Algorithm during Straight Walking and Turning in Patients with Parkinson’s Disease and Older Adults Using an Inertial Measurement Unit at the Lower Back. Front. Neurol..

[31] Shin S.H., Park C.G. (2011). Adaptive step length estimation algorithm using optimal parameters and movement status awareness. Med. Eng. Phys..

[32] Weinberg H. (2002). Using the ADXL202 in Pedometer and Personal Navigation Applications. Analog. Devices 602 Appl. Note.

[33] Bylemans I., Weyn M., Klepal M. (2009). Mobile phone-based displacement estimation for opportunistic localisation systems. 2009 Third International Conference on Mobile Ubiquitous Computing, Systems, Services and Technologies.

[34] Bohannon R.W., Glenney S.S. (2014). Minimal clinically important difference for change in comfortable gait speed of adults with pathology: A systematic review. J. Eval. Clin. Pract..

[35] Bonci T., Keogh A., Del Din S., Scott K., Mazzà C., on behalf of the Mobilise-D consortium (2020). An Objective Methodology for the Selection of a Device for Continuous Mobility Assessment. Sensors.

[36] Brand Y.E., Buchman A.S., Kluge F., Palmerini L., Becker C., Cereatti A., Maetzler W., Vereijken B., Yarnall A.J., Rochester L. (2026). Continuous assessment of daily-living gait using self-supervised learning of wrist-worn accelerometer data. npj Digit. Med..

[37] Mirelman A., Bernad-Elazari H., Nobel T., Thaler A., Peruzzi A., Plotnik M., Giladi N., Hausdorff J.M. (2015). Effects of Aging on Arm Swing during Gait: The Role of Gait Speed and Dual Tasking. PLoS ONE.

