# Supplementary material for: Validation of an Algorithmic Pipeline for Wrist-Worn Devices to Estimate Walking Speed in People with Multiple Long-Term Conditions
Source: Sensors (Basel). 2026 Aug 5;26(15):4946. doi: 10.3390/s26154946 (PMC13468760; doi:10.3390/s26154946)
Supplement: Supplementary file 1 [file sensors-26-04946-s001.zip › sensors-4448258-supplementary.pdf]

Supplementary Material to:

Validation of an algorithmic pipeline for wrist-worn devices to estimate walking speed in people with multiple long-term conditions

Dimitrios Megaritis, Lisa Alcock, Kirsty Scott, Hugo Hiden, Ioannis Vogiatzis, and Silvia Del Din

Table of Contents

Methods **2**

Pipeline Pseudocode **2**

Performance Indices **2**

Results **6**

Reference list **11**

Methods

Pipeline Pseudocode

Algorithm S1: Pipeline process

Input: Raw wrist IMU signal I (tri-axial acceleration; 100 Hz)

Output: WB-level cadence, stride length (SL), and walking speed (WS)

```
procedure ESTIMATE_DMOs(I)
  GS_list ← GSD(I)   // detects candidate gait sequences
  WB_results ← ∅
  for each GS in GS_list do
    IC_list ← ICD(GS)   // detects initial contacts within each GS
    if |IC_list| < 2 then
      discard GS   // insufficient contacts for stride-level estimation
      continue
    end if
    Cad_sec ← CADENCE_ESTIMATE(GS, IC_list)   // per-second cadence
    SL_sec ← SL_ESTIMATE(GS, IC_list)   // per-second stride length
    WS_sec ← (Cad_sec × SL_sec) / 120   // per-second walking speed
    Cad_stride, SL_stride, WS_stride ← SEC_TO_STRIDE(Cad_sec, SL_sec, WS_sec, IC_list)
      // maps per-second values onto per-stride regions
    Cad_WB, SL_WB, WS_WB ← MEAN(Cad_stride), MEAN(SL_stride), MEAN(WS_stride)
      // per-stride values averaged over the GS/WB window
    WB_results ← WB_results ∪ {GS, Cad_WB, SL_WB, WS_WB}
  end for
  return WB_results
end procedure
```

Note: GSD, ICD, CADENCE_ESTIMATE, and SL_ESTIMATE denote the best-performing wrist-adapted algorithm variants selected per block; full algorithmic detail and implementations are provided in the accompanying open-source repository.

Performance Indices

Performance indices were calculated following the weighted decision matrix approach described in Bonci et al. [1] and adapted previously by Megaritis [2] and Micó -Amigo [3]. Briefly, each metric was classified as either a benefit (higher values indicate better performance) or a cost (lower values indicate better performance). Cost metrics were normalised to a 0–1 scale using exponential normalisation, with the exception of absolute cadence error which used min-max normalisation. Benefit metrics were used directly. A weighted sum of all normalised metrics produced a performance index ranging from 0 (worst) to 1 (best).

For Aim 1 (GSD, ICD, SL), metric weights follow those previously reported and validated [2], presented in Tables S1–S3. For Aim 2, cadence performance index weights follow those

previously reported in Micó-Amigo et al. [3], while a novel performance index was developed for walking speed estimation, with weights for both presented in Tables S4–S5.

Table S1 . GSD performance index weights

Metric	Classification	Weight
Absolute relative duration error	Cost	0.185
ICC(2,1)	Benefit	0.155
Specificity	Benefit	0.203
Accuracy	Benefit	0.185
Recall (Sensitivity)	Benefit	0.142
Precision (Positive Predictive Value)	Benefit	0.130

Table S2 . ICD performance index weights

Metric	Classification	Weight
Recall (Sensitivity)	Benefit	0.237
Precision (Positive Predictive Value)	Benefit	0.229
Absolute timing error (s)	Cost	0.267
Relative timing error	Cost	0.267

Table S3 . SL performance index weights

Metric	Classification	Weight
Absolute error (m)	Cost	0.277
Relative error	Cost	0.277
ICC(2,1)	Benefit	0.446

Table S4 . CAD performance index weights

Metric	Classification	Weight
Absolute error (steps/min)	cost	0.277
Relative error	cost	0.277
ICC(2,1)	benefit	0.446

Table S5 . WS performance index weights

Metric	Classification	Weight
ICC(2,1)	Benefit	0.223
Absolute error (m/s)	Cost	0.277
Limits of agreement (m/s)	Cost	0.223
Relative error	Cost	0.277

Note that for the participant-level analysis (Aim 3), simplified performance indices were computed excluding ICC and limits of agreement, with remaining metric weights redistributed proportionally. These are presented in Tables S1–S5 in the online supplement.

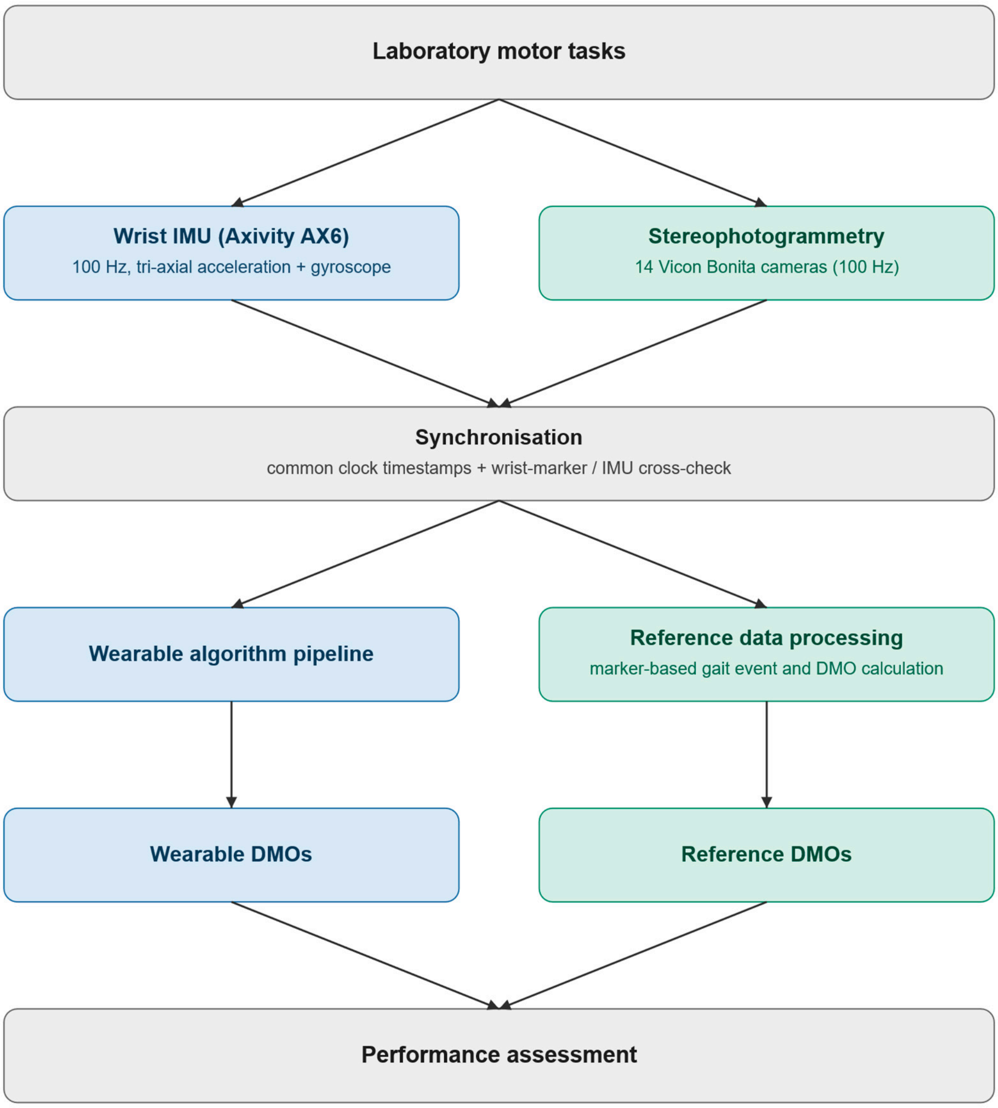

Figure S1. Overview of the data acquisition and processing pipeline, showing the two synchronised data streams (wrist-worn IMU and stereophotogrammetry) recorded during laboratory tasks, their independent processing into wearable and reference digital mobility outcomes (DMOs), and the subsequent performance assessment.

Algorithm S2: Performance index calculation and algorithm ranking

Input: Per-block metric values M = {m_1, ..., m_K} across all WBs/tests for algorithm A; weighting scheme W = {(criterion_k, method_k, weight_k)}

Output: Performance Index PI(A); ranking of candidate algorithms

```
procedure COMPUTE_PI(M, W)
  PI ← 0
  for each metric_k in W do
    x ← M[metric_k]   // raw per-WB/per-test values
    if metric_k = ICC then
      e_k ← ICC(2,1)(x)
      e_k ← clip((e_k + 1) / 2, 0, 1)   // rescale [-1,1] to [0,1]
    else if metric_k = LoA then
      width ← 2 × 1.96 × SD(x)   // Bland-Altman limits of agreement width
      e_k ← exp(−width)
    else
      e_k ← NORMALISE(x, criterion_k, method_k)
      e_k ← MEAN(e_k)   // averaged across WBs/tests
    end if
    PI ← PI + weight_k × e_k
  end for
  return PI
end procedure
function NORMALISE(x, criterion, method)
  if method = exponential then
    x_norm ← 1 − exp(−x)
  else if method = minmax then
    x_norm ← (x − min(x)) / (max(x) − min(x))
  else
    x_norm ← x   // benefit metrics, used directly
  end if
  if criterion = cost then
    return 1 − x_norm   // = exp(−x) when method = exponential
  else
    return x_norm
  end if
end function
procedure RANK(candidates, W)
  for each algorithm A in candidates do
    PI(A) ← COMPUTE_PI(M_A, W)
  end for
  return candidates sorted by PI descending
end procedure
```

Note: ICC denotes the intraclass correlation coefficient (2,1); LoA denotes limits of agreement, computed as mean error ± 1.96×SD; NORMALISE implements the cost/benefit and exponential/min-max normalisation scheme underlying Tables S1–S5.

Results

Table S6 . Stride length algorithm performance metrics evaluated using reference-derived walking bouts and initial contacts, ranked by performance index, with all metrics reported as mean values with 95% confidence intervals [95% CI].

Algorithm	Performance Index	n WBs	Absolute Error (m)	ICC
Weinberg*	0.80	561	0.09 [0.07, 0.11]	0.83 [0.72, 0.90]
Weinberg	0.78	561	0.10 [0.08, 0.12]	0.80 [0.68, 0.88]
Bylemans*^	0.74	561	0.14 [0.10, 0.17]	0.68 [0.54, 0.79]
Weinberg*^	0.72	561	0.12 [0.09, 0.15]	0.64 [0.50, 0.76]
Weinberg^	0.72	561	0.12 [0.10, 0.15]	0.62 [0.48, 0.74]
Bylemans	0.69	561	0.14 [0.10, 0.19]	0.61 [0.45, 0.76]
Bylemans*	0.68	561	0.23 [0.16, 0.30]	0.62 [0.46, 0.76]
Bylemans^	0.68	561	0.14 [0.10, 0.17]	0.55 [0.42, 0.69]
Kim*	0.68	561	0.15 [0.12, 0.19]	0.55 [0.45, 0.66]
Kim*^	0.67	561	0.16 [0.12, 0.20]	0.51 [0.40, 0.64]
Kim	0.66	561	0.18 [0.14, 0.23]	0.50 [0.42, 0.61]
Kim^	0.66	561	0.17 [0.13, 0.21]	0.50 [0.41, 0.62]

ICC: intraclass correlation coefficient; N WBs: number of reference walking bouts; 95% CI: 95% confidence interval. Reference-derived walking bouts and initial contacts were provided as inputs to isolate standalone SL algorithm performance from pipeline effects. *Adaptive version; ^foot length-augmented version.

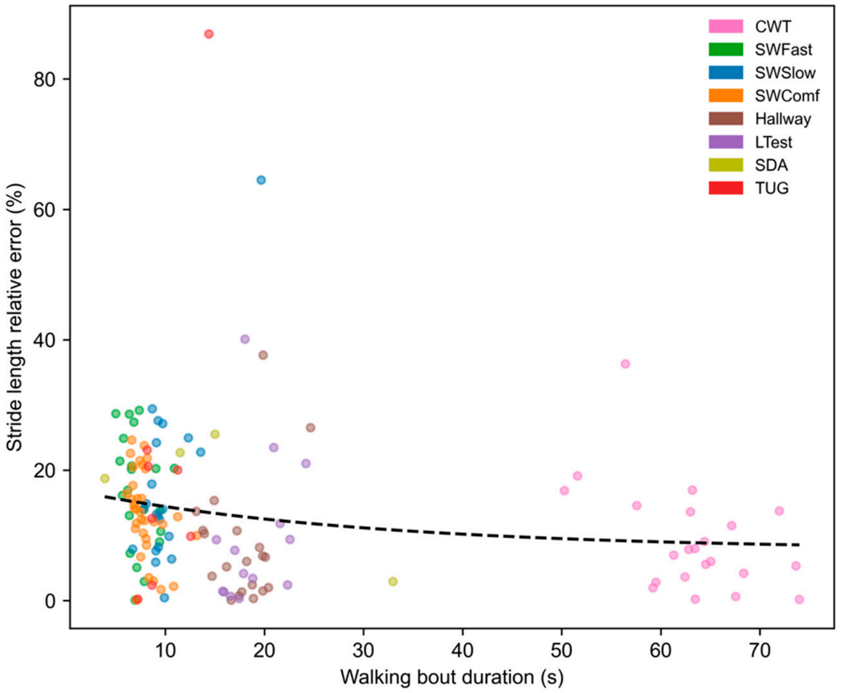

Figure S2 . Stride length relative error versus walking bout duration for the best-performing pipeline (IonescuGSD + ZijlstraIC + WeinbergSL adaptive) across all true positive walking bouts. The dashed line represents an exponential decay fit. Each point represents one walking bout, colour-coded by laboratory task. SW: straight walking; Comf: comfortable speed; Slow: slow speed; Fast: fast speed; TUG: Timed Up and Go; LTest: L-shaped walking test; CWT: Continuous Walking Test; SDA: Simulated Daily Activities.

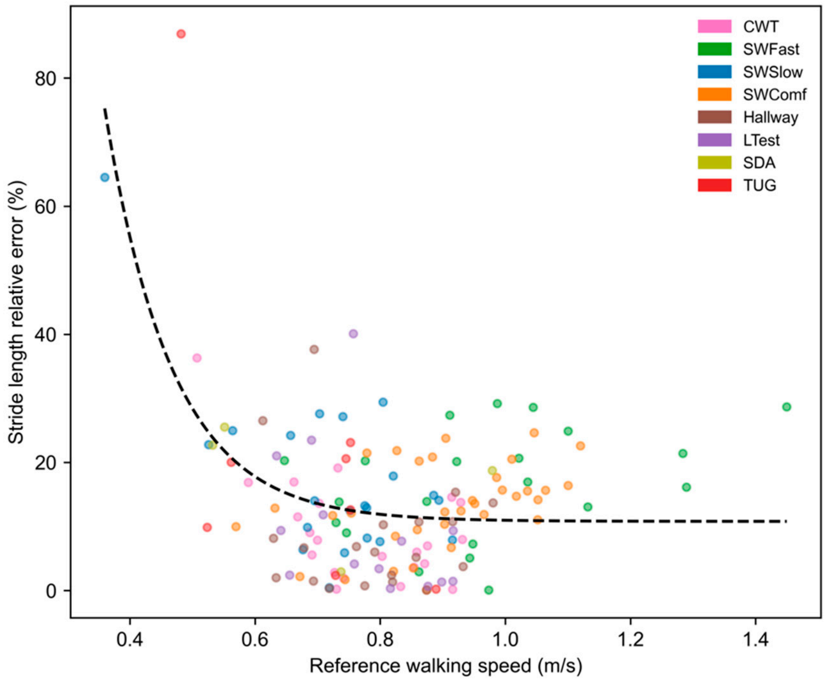

Figure S3 . Stride length relative error versus reference walking speed for the best-performing pipeline (Ionescu + Zijlstra + Weinberg adaptive) across all true positive walking bouts. The dashed line represents an exponential decay fit. Each point represents one walking bout, colour-coded by laboratory task. SW: straight walking; Comf: comfortable speed; Slow: slow speed; Fast: fast speed; TUG: Timed Up and Go; LTest: L-shaped walking test; CWT: Continuous Walking Test; SDA: Simulated Daily Activities.

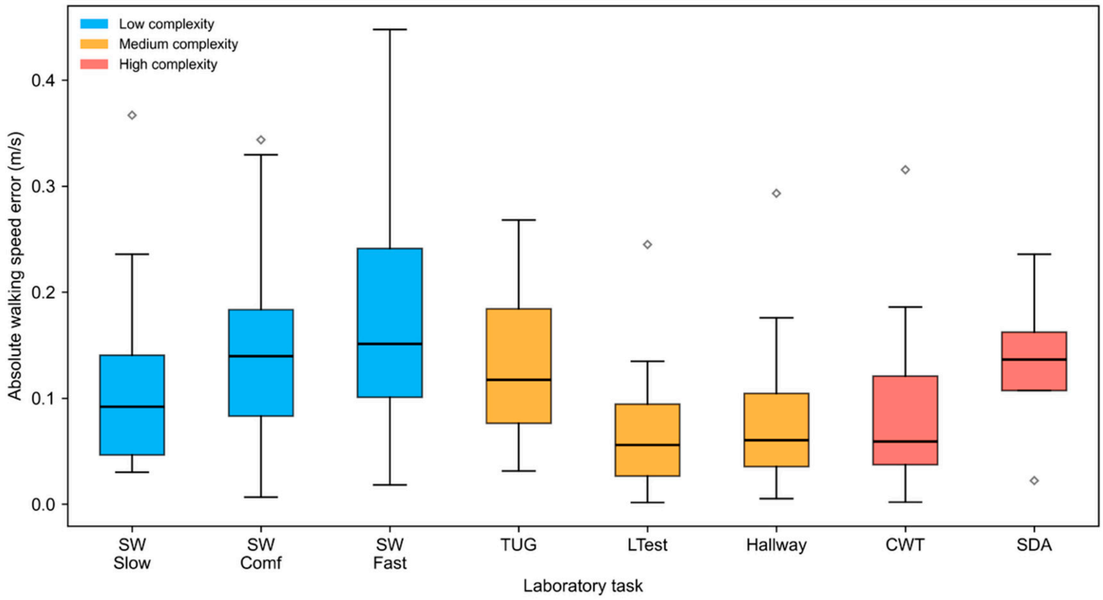

Figure S4 . Absolute walking speed error by laboratory task for the best-performing pipeline (Ionescu + Zijlstra + Weinberg adaptive) across all true positive walking bouts. Tasks are grouped and colour-coded by complexity: low complexity (blue), medium complexity (orange), and high complexity (red). The horizontal black line represents the median, boxes represent the interquartile range, whiskers extend to 1.5× IQR, and diamonds represent outliers.

Table S7 . Mean and median percentage performance loss from the population-best modular pipeline by number of multimorbidity clusters.

		Overall		GSD		ICD		Cadence		SL		WS	
n_clusters	N	Mean	Median	Mean	Median	Mean	Median	Mean	Median	Mean	Median	Mean	Median
1	4	6.44	5.95	5.82	5.82	5.23	4.15	1.76	1.29	8.74	6.75	10.68	10.81
2	21	4.77	4.2	6.54	1.09	5.21	5.23	1.29	0.79	5.87	4.06	4.95	4.54
3	14	3.79	3.11	2.6	0	4.57	4.5	2.06	1.42	3.84	3.43	5.86	3.78
4	6	4.13	4.2	7.63	1.15	5.25	3.27	0.67	0.4	2.49	2.93	4.61	3.57

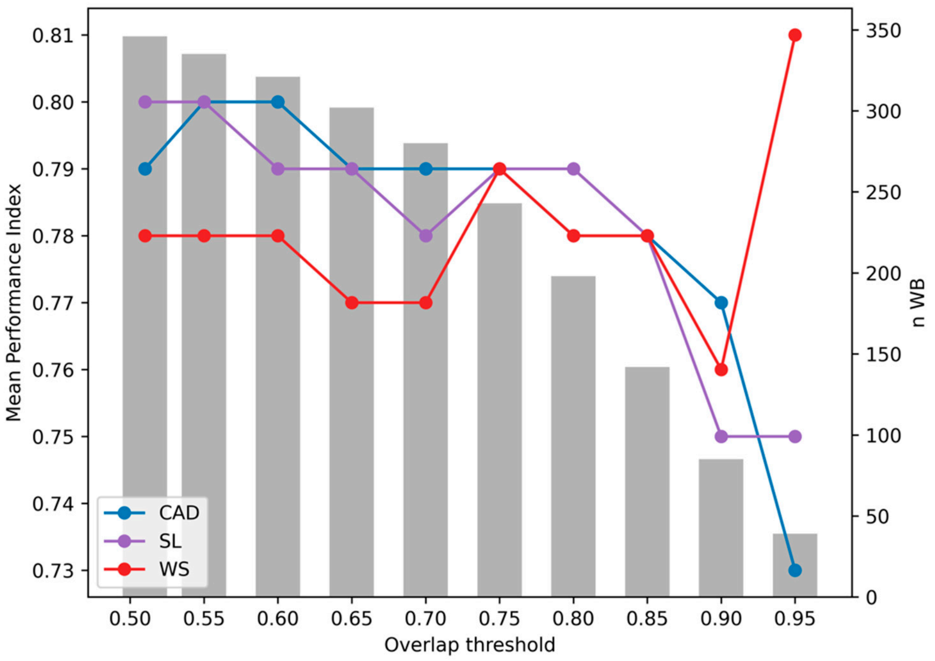

Figure S5 . Mean performance index for cadence (CAD), stride length (SL), and walking speed (WS) as a function of the GSD overlap threshold used to define true positive walking bouts, using the finalised algorithms from the modular population-best pipeline. Grey bars show the number of walking bouts retained at each threshold (n WB, right axis).

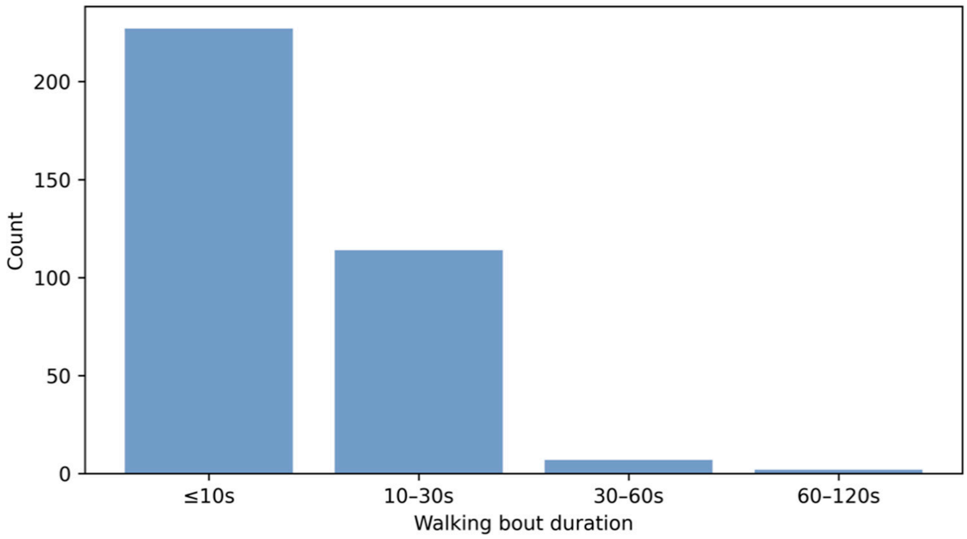

Figure S6 . Distribution of walking bout durations for wearable-detected bouts excluded from the true positive (TP) evaluation (overlap ≤85% with the reference system).

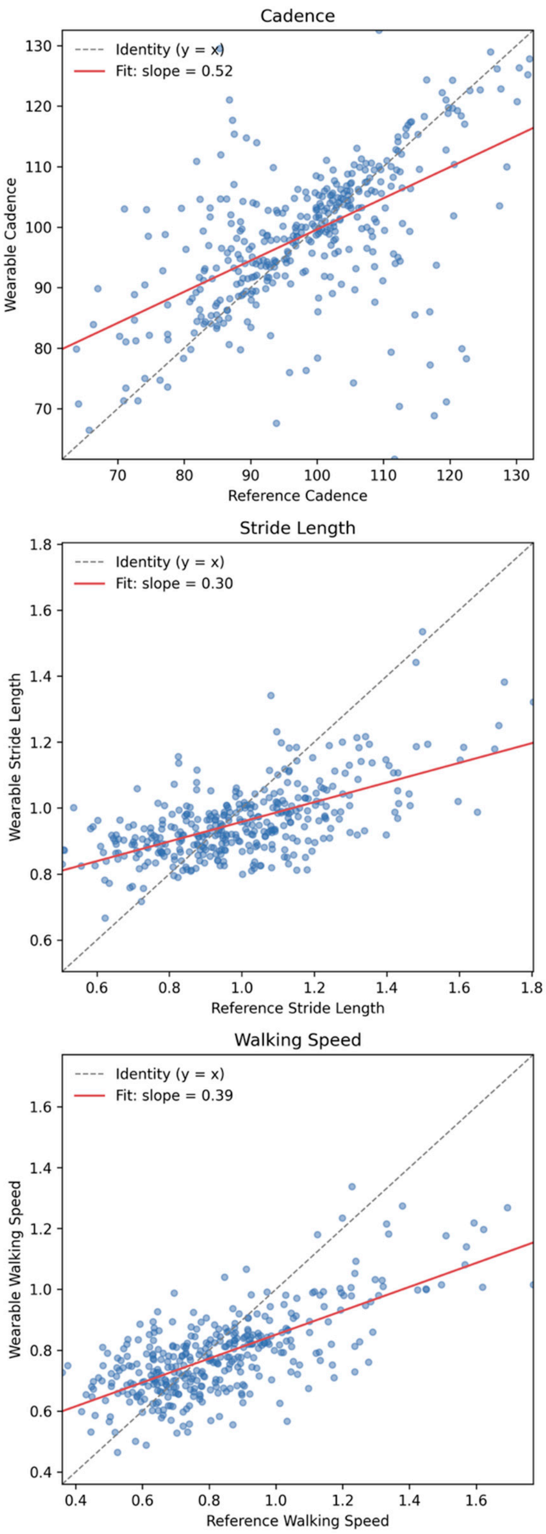

Figure S7 . Scatter plots of wearable-derived versus reference-derived (A) cadence, (B) stride length, and (C) walking speed values across all laboratory tasks (complete-case analysis; nWB = 504). Dashed line indicates identity (y = x); solid line indicates the fitted linear regression, with slope reported for each panel.

Reference list

1. Bonci, T., et al. An Objective Methodology for the Selection of a Device for Continuous Mobility Assessment. Sensors, 2020. **20**, 6509 DOI: 10.3390/s20226509.
2. Megaritis, D., et al., Real-World Wrist-Derived Digital Mobility Outcomes in People with Multiple Long-Term Conditions: A Comparison of Algorithms. Bioengineering (Basel), 2025. **12**(10).
3. Micó-Amigo, M.E., et al., Assessing real-world gait with digital technology? Validation, insights and recommendations from the Mobilise-D consortium. Journal of NeuroEngineering and Rehabilitation, 2023. **20**(1): p. 78.
